# Novel Chlorin Derivatives with a Modified Exocycle E: A Study of Spectral and Biological Properties

**DOI:** 10.3390/ijms27219845

**Published:** 2026-11-03

**Authors:** Viktor A. Pogorilyy, Julia S. Kartava, Tanaka F. Tandare, Victoria V. Shchelkova, Nikita V. Suvorov, Yuriy L. Vasil’ev, Sergey S. Dydykin, Ivan Varga, Michael A. Grin

**Affiliations:** 1 Institute of Fine Chemical Technologies, MIREA-Russian Technological University, 86 Vernadsky Avenue, Moscow 119571, Russia; takerroy@yandex.ru (T.F.T.); victoria.shchelkova@yandex.ru (V.V.S.); suvorov.nv@gmail.com (N.V.S.); y_vasiliev@list.ru (Y.L.V.); michael_grin@mail.ru (M.A.G.); 2 Russian State University Named After A. N. Kosygin (Technologies. Design. Art), str Malaya Kaluzhskaya 1, Moscow 115035, Russia; 3 Department of Operative Surgery and Topographic Anatomy, I.M. Sechenov First Moscow State Medical University ( Sechenov University), str Trubetskaya 8\2, Moscow 119435, Russia; dydykin_s_s@staff.sechenov.ru (S.S.D.); ivan.varga@fmed.uniba.sk (I.V.); 4 Institute of Histology and Embryology, Faculty of Medicine, Comenius University in Bratislava, Sasinkova 4, 811 08 Bratislava, Slovakia

**Keywords:** antimicrobial photodynamic therapy (aPDT), photodynamic therapy (PDT), photosensitizers (PSs), natural chlorin, purpurinimide, cyclic amine, thioimide

## Abstract

For the first time, new natural chlorin derivatives containing tetrahydropyridine and thioimide exocycles have been synthesized. The spectral characteristics of the synthesized compounds were studied: for the thioimide, a bathochromic displacement of the long-wave absorption maximum (by 33 nm) is shown, and the tetrahydropyridine derivative has a hypsochromic displacement of the *Q*_2_-band (by 52 nm) compared to the original purpurinimide. For the resulting sulfur-containing derivative, an increase in the quantum yield of singlet oxygen generation with almost complete fluorescence quenching was found, while for the tetrahydropyridine, singlet oxygen generation was reduced by half, while fluorescence increased five-fold. Both synthesized compounds had a high phototoxicity activity for both Gram-positive and Gram-negative strains of bacteria, which indicates their potential as photosensitizers for antimicrobial photodynamic therapy.

## 1. Introduction

Antimicrobial photodynamic therapy (aPDT) is one of the applied fields of PDT aimed at the destruction of pathogenic microorganisms. Antimicrobial PDT is a promising alternative to antibiotic therapy, as it does not cause resistance and is highly effective against a wide range of pathogens [1,2,3]. However, the restriction of the use of aPDT is associated with the depth of light penetration into tissue when irradiating infected foci, the stability and effectiveness of the photosensitizers (PSs) used, as well as the selectivity of their action [4,5].

A wide range of compounds of various classes has been studied as potential photosensitizers for aPDT [6,7,8,9,10,11]. Among them, special attention is drawn to fluorophores of natural origin based on tetrapyrrole macroheterocycles, which are viewed as the most promising agents for combatting localized infections. In particular, chlorins exhibit intense absorption bands in the red and near-infrared regions of the electromagnetic spectrum, which ensures a deeper penetration of radiation into biological tissues [12,13]. Photosensitizers based on natural chlorins and their derivatives exhibit high activity against a broad range of microorganisms, including Gram-positive and Gram-negative bacteria and pathogenic fungi. Their potential for the treatment of bacterial biofilms has also been reported [14,15,16,17,18,19,20,21,22].

Commercial drugs based on chlorin *E*_6_, used in the photodynamic therapy of oncological diseases, are also studied as PSs to combat bacterial infections [23,24,25]. However, they have a disadvantage associated with limited accumulation in Gram-negative strains, since under physiological conditions they are in anionic form [26]. Therefore, there is a need to develop new agents for aPDT with improved accumulation selectivity in bacterial cells.

One of the most promising classes of compounds as PSs for the photodynamic therapy of cancer are natural chlorin derivatives based on purpurinimides, which can also be effective for aPDT [27,28,29,30,31]. Due to the presence of a nitrogen atom in exocycle E, which can be protonated under physiological conditions into a cationic form, the latter can better penetrate through the membranes of bacterial cells, thereby showing greater effectiveness against Gram-negative bacteria compared to known PSs [32,33,34,35].

However, the presence of two carbonyl groups in the exocycle E can lead to interactions with non-target biological components of cells, which will contribute to reducing the stability and increasing the toxicity of these agents in normal tissues. In this regard, this work implemented a bioisosteric substitution [36,37,38,39], often used in medical chemistry to eliminate unwanted interactions, including the synthesis of thiocarbonyl derivatives [40,41,42,43,44].

Another approach involves the reduction of carbonyl groups in the exocycle E, which contributes to the prevention of aggregation, the increase in quantum fluorescence yields, the generation of reactive oxygen species (ROS), and the improvement of the pharmacokinetic profile of a PS [45,46,47,48]. Replacing oxygen with sulfur promotes intersystem crossing, which causes a bathochromic shift of the long-wavelength absorption maxima into the near-infrared region (NIR) of the spectrum and an increase in singlet oxygen generation [49,50,51,52].

Our research group has extensive experience in the synthesis of sulfur-containing derivatives of natural chlorins—thioanhydrides, thiocarbonyl compounds and their analogs. The resulting compounds are characterized by high quantum yield of singlet oxygen generation and significant photoinduced cytotoxicity [53,54,55,56]. Accordingly, we synthesized the first representatives of two new compound classes of purpurinimide derivatives, including tetrahydropyridine and purpurinthioimide derivatives, which we also studied in this work.

This study presents the synthesis and characterization of previously undescribed reduced and thioimide derivatives of a natural chlorin and elucidates their distinct structural, photophysical, and photodynamic properties. In particular, the work reveals novel chemistry in the tetrahydropyridine fragment formed upon reduction of the exocyclic E-ring, demonstrating that this structural modification provides a new pathway for the functionalization of the chlorin macrocycle. The corresponding thioimide derivative constitutes another unexplored modification of this structural motif and enables comparison of the effects of oxygen- and sulfur-containing functionalities on the electronic and photophysical properties of the photosensitizer.

## 2. Results

### 2.1. Synthesis of the Tetrahydropyridine Natural Chlorin Derivative

**Scheme 1:**
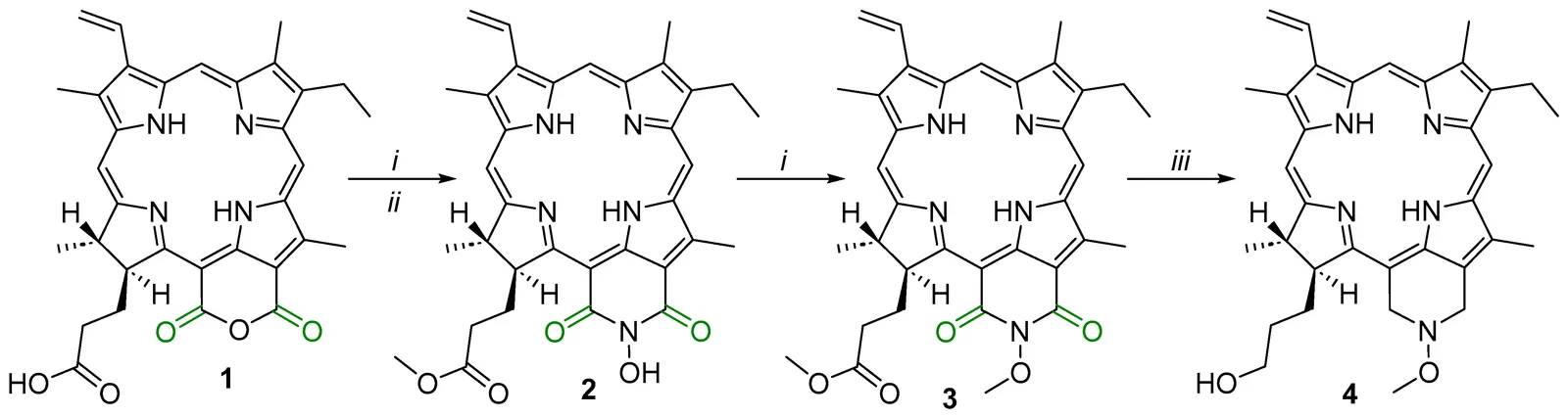
Synthesis of *N*-methoxypurpurinimide-18 (**3**) and the reduction reaction of exocycle E. Reaction conditions and reagents: *i*—CH_2_N_2_, (Et)_2_O, CH_2_Cl_2_, 0 °C, 1 h; *ii*—hydroxylamine hydrochloride (NH_2_OH • HCl), pyridine, 20 °C, 12 h; *iii*—LiAlH_4_, AlCl_3_, THF, 0 °C, 2 h.

This paper proposes an approach for the reduction of the exocycle in *N*-hydroxypurpurinimide (**2**). This compound was obtained from the methyl ester of purpurin-18 (**1**) according to the method described earlier [57,58]. The synthesis of the starting compound purpurin-18 (**1**) was carried out from naturally derived *Spirulina platensis* biomass as the initial raw material by extraction in acetone [59], performed similarly to the method described in the literature [60]. To prevent adverse reactions of the hydroxyl group at the nitrogen atom of exocyclic ring E, we obtained a derivative of compound (**2**) containing an ether bond, namely *N*-methoxypurpurinimide-18 (**3**). The reaction of the imide cycle of this compound with lithium aluminum hydride (LiAlH_4_) in THF and the addition of two equivalents of aluminum chloride (AlCl_3_) led to the reduction of two carbonyl groups of the exocycle E and the ester substituent in position 17^3^. Thus, during this work, chlorin (**4**) containing a tetrahydropyridine fragment was obtained for the first time (Scheme 1).

The structure of the obtained derivative (**4**), containing the tetrahydropyridine cycle, was confirmed by a combination of physicochemical methods of analysis. In the ^1^H NMR spectrum, a broad multiplet in the region of 5.87–4.90 ppm was observed, which was attributed to hydrogen atoms in positions 13^1^ and 15^1^ of the reduced exocycle E (Figure S2).

Low energy barriers for large amplitude movements, such as nitrogen inversion and cycle pseudoinversion, explain the broadening of ^1^H NMR signals in the reduced exocycle. These processes cause rapid conformational exchange under experimental conditions, which dynamically averages the proton environments and leads to widening of resonance lines, especially for protons close to nitrogen or in sterically sensitive positions [61].

### 2.2. Synthesis of the Thiocarbonyl Chlorin Derivative

**Scheme 2:**
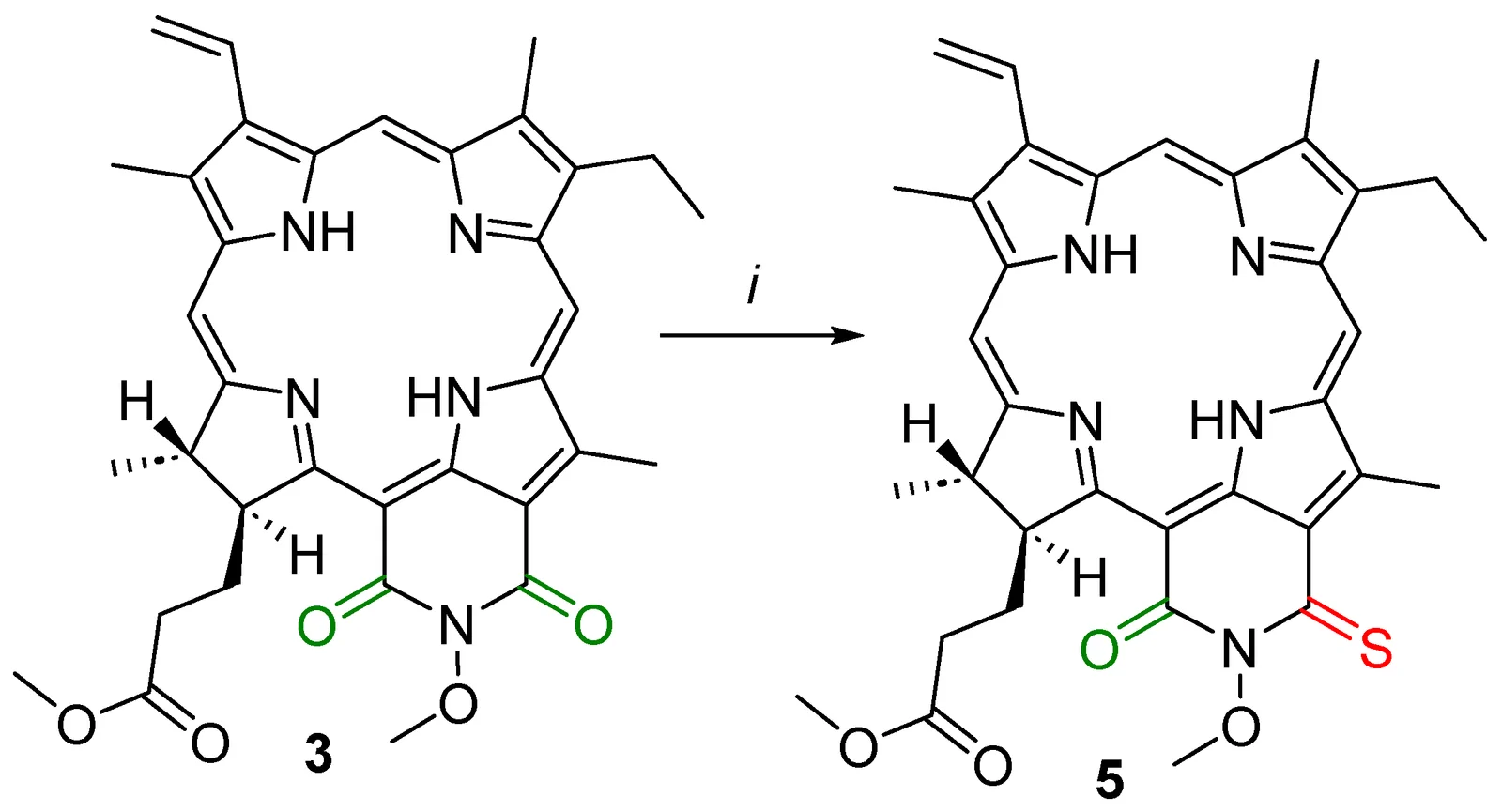
Synthesis of monothioimide (**5**): *i*—Lawesson’s reagent, toluene, 110 °C, 6 h, Ar.

In this work, a thioimide derivative (**5**) in the purpurinimide class was obtained for the first time by the reaction of hiolation of the methyl ester of *N*-methoxypurpurinimide (**3**) using Lawesson’s reagent. The substitution of the oxygen atom with sulfur occurred along the carbonyl group in position 13^1^ of the exocycle due to its greater steric availability (Scheme 2).

On the ^1^H NMR spectrum of the compound (**5**) (Figure S5), a downfield shift was observed for the signal corresponding to the methyl protons at position 12 of the macrocycle compared to the original compound (**3**) and on the ^13^C NMR spectrum (Figure S6)—a downfield shift was observed for the signal assigned to C-13^1^ of the macrocycle [62], which is consistent with the structure of the resulting methyl ester of *N*-methoxypurpurinimide (**5**), namely with the substitution of the oxygen atom with sulfur in the exocycle E in position 13^1^.

### 2.3. Study of the Spectral and Photophysical Properties of the Synthesized Compounds

**Figure 1:**
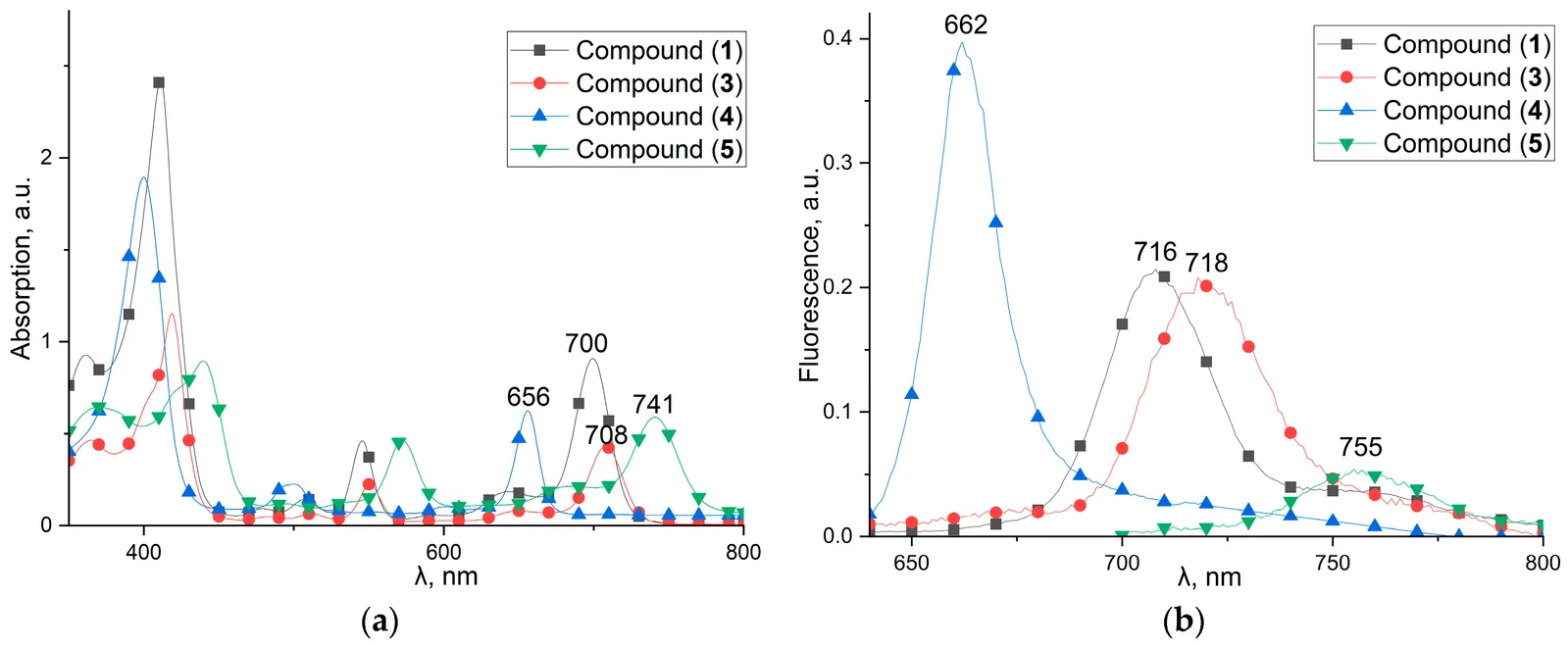
(**a**) The electronic absorption spectra of compound (**1**) and its derivatives (**3**)–(**5**) in dichloromethane. (**b**) The electronic fluorescence spectra of the compound (**1**), (**3**)–(**5**) in dichloromethane.

The photophysical properties of the resulting compounds were studied, including the electronic absorption and fluorescence spectra, which were obtained in dichloromethane (Figure 1) and a 4% micellar solution of Kolliphor^®^ ELP (Figure S7). The quantum fluorescence yield and the quantum yield of singlet oxygen generation were also measured (Table 1, Figures S8 and S9).

On the electronic absorption spectra in dichloromethane for compound (**4**), a hypsochromic shift in the maximum of 52 nm was observed, and for compound (**5**)—a bathochromic shift of 33 nm compared to the original compound (**3**) (Figure 1a). The resulting thiocarbonyl derivative of *N*-methoxypurpurinimide (**5**) had an absorption maximum at 741 nm, which corresponds to the characteristic absorption range of bacteriochlorins.

The fluorescence spectra were recorded using an excitation wavelength of 530 nm (Figure 1b). The fluorescence quantum yield for all compounds was calculated according to a known method [63]. The quantum fluorescence yield in dichloromethane for compound (**5**) was 5.4 times lower than that of the starting compound *N*-methoxypurpurinimide (**3**) (0.049), and 5.1 times higher for compound (**4**).

Measurements of quantum yields of singlet oxygen generation were carried out according to a modified version of a previously reported method [64,65]. The values of this parameter in dichloromethane for compound (**5**) remained almost the same, and for compound (**4**) decreased by 1.84 times compared to the original purpurinimide (**3**).

In the aqueous micellar medium, no aggregation was observed: in the absorption spectra recorded over a wide concentration range, the profile of the absorption maximum bands remained unchanged—without broadening or shifts, and the absorption intensity strictly obeyed the Bouguer–Lambert–Beer law (Figure S10). Dynamic light scattering (DLS) data demonstrated a monodisperse particle size distribution corresponding to the hydrodynamic diameter of individual micelles, with no formation of large aggregates (Figure S11). Photostability was assessed during the singlet oxygen generation experiments: all compounds remained stable upon irradiation at their absorption maxima for 10 min.

### 2.4. Quantum-Chemical Calculations of Energy Levels of Molecular Orbitals Using the DFT Method

Quantum chemical calculations of natural chlorin molecules were performed using the density functional theory (DFT) and time-dependent DFT (TD-DFT) methods in the ORCA 6.0.1 software package. The geometry of the molecules was pre-optimized, after which the energy levels of molecular orbitals (MOs) were calculated.

The def2-TZVP basis set was used in combination with the PW6B95 exchange-correlation functional. These computational parameters were selected to achieve an optimal balance between the accuracy of the electronic-structure description and computational cost, which is particularly important given the large number of atoms in the natural chlorin molecules [66,67].

**Figure 2:**
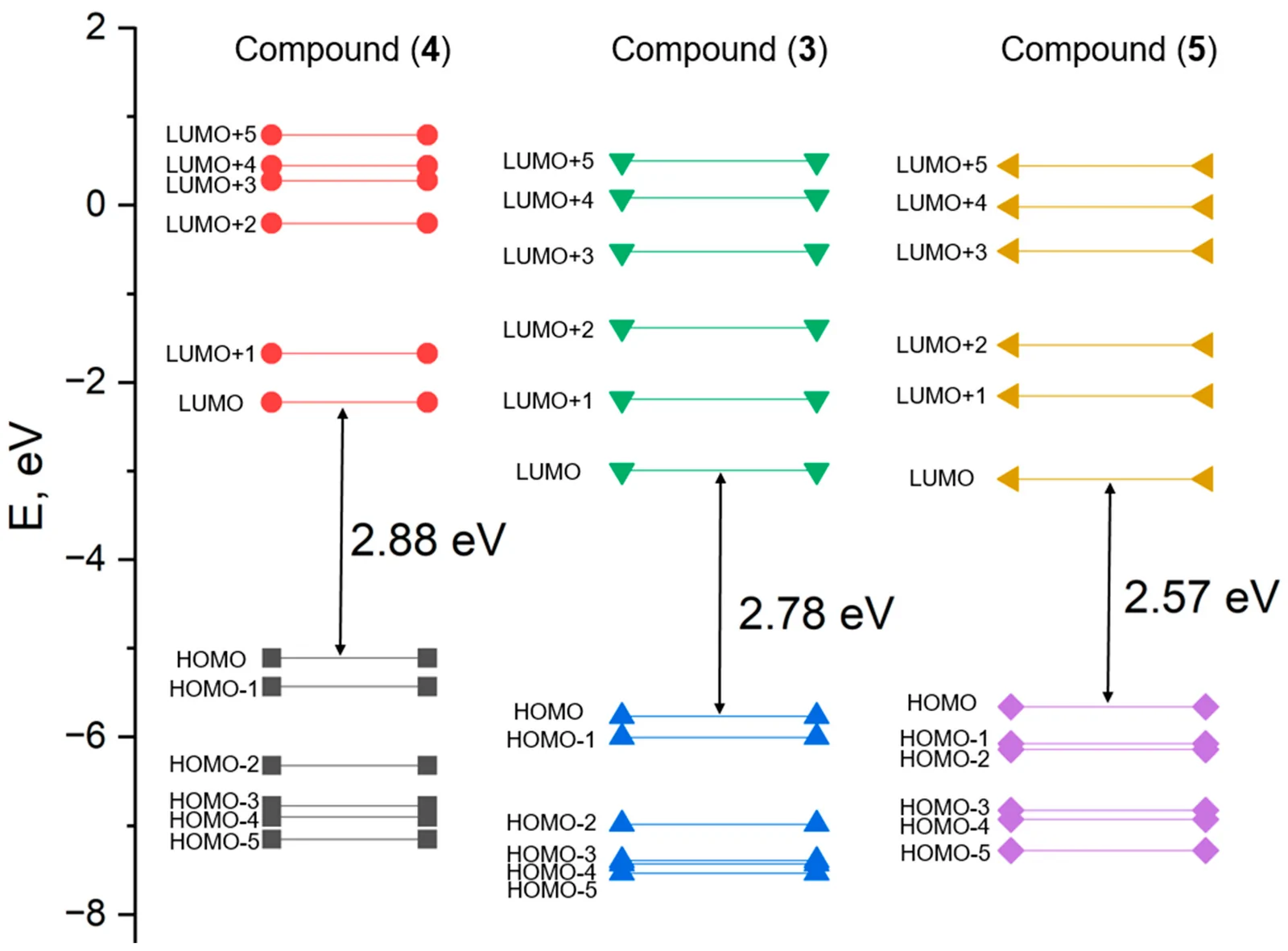
Kohn–Sham (eV) MO energy level diagram for compounds (**3**), (**4**) and (**5**) def2-TZVP and PW6B95.

As a result of studies of the energy levels of the MOs, the Kohn–Sham diagram (eV) was constructed for three compounds (**3**), (**4**) and (**5**) presented in Figure 2.

### 2.5. Experimental Assessment of Photoinduced and Dark Bactericidal Activity of the Obtained Compounds In Vitro

In this study, the biological activity of the test compounds was initially evaluated in vitro using planktonic bacterial cultures.

Studies of the biological antimicrobial activity of tetrahydropyridine (**4**) and thioimide (**5**) chlorin derivatives were carried out on two model strains: Gram-positive *Staphylococcus aureus* and Gram-negative *Pseudomonas aeruginosa*. *N*-methoxypurpurinimide (**3**) was selected as the reference compound. The compounds were solubilized in aqueous medium by incorporation into micelles formed with 4% Kolliphor^®^ ELP following a method previously developed by our research group [55].

**Figure 3:**
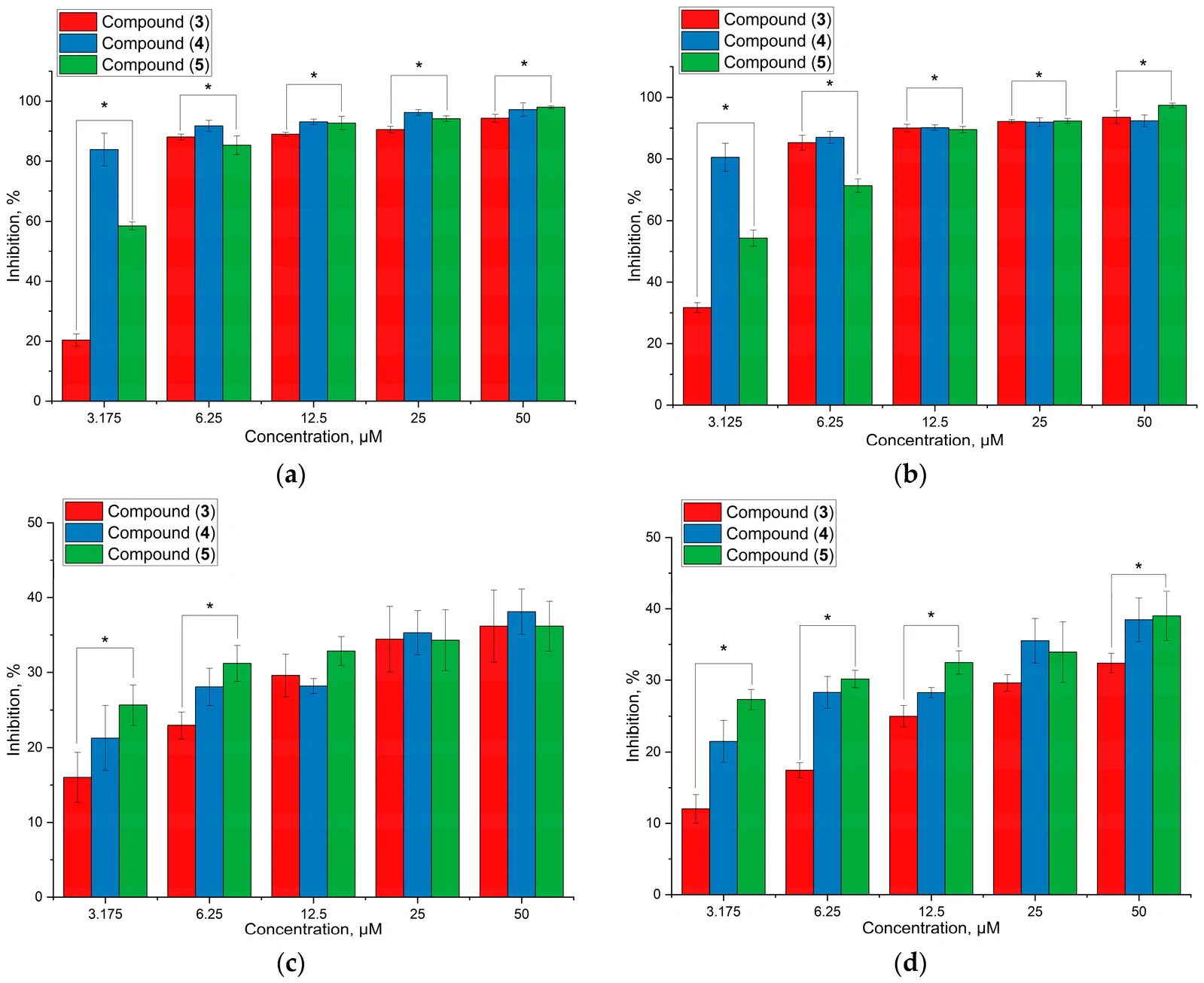
Photoinduced (**a**,**b**) and dark (**c**,**d**) cytotoxicity of compounds (**3**)–(**5**) against *S. aureus* (**a**,**c**) and *P. aeruginosa* (**b**,**d**); *—a statistically significant difference in absorption between the groups at *p* < 0.05.

Incubation of bacterial cells with the studied compounds led to the suppression of their growth both during irradiation and in its absence, depending on the concentration (Figure 3, Table S4). At the lowest tested concentration (3.175 μM), the new purpurinimide derivatives (**4**) and (**5**) exhibited superior antibacterial activity both photoinduced and dark relative to the parent compound (**3**). Specifically, their inhibition percentages were 2–4 times higher upon irradiation and 1.5–2 times higher in the dark for both bacterial strains. At higher concentrations under illumination, derivatives (**4**) and (**5**) showed activity comparable to that of compound (**3**). In the absence of light at 25–50 μM a similar trend was observed for the Gram-positive strain *S. aureus*; for *P. aeruginosa*, however, the inhibition by derivatives (**4**) and (**5**) was approximately 1.2-fold higher.

Overall, these results indicate that derivatives (**4**) and (**5**) exhibit enhanced antibacterial activity at low concentrations, particularly under irradiation, highlighting their potential as more effective photosensitizers compared with the parent compound (**3**).

## 3. Discussion

### 3.1. Synthesis of the Tetrahydropyridine Natural Chlorin Derivative

*N*-methoxypurpurinimide methyl ester (**3**) was synthesized from purpurin-18 (**1**) [59] following a procedure in the literature [57,58]. Owing to its pronounced hydrophilicity and the consequent difficulties encountered in its purification, *N*-hydroxypurpurinimide (**2**) was not isolated from the reaction mixture, and compound (**3**) was obtained directly from (**2**) in situ.

The proposed approach to the synthesis of compound (**4**) proved to be effective for the selective reduction of exocyclic ring E. The key step was the preliminary protection of the *N*-hydroxyl group of *N*-hydroxypurpurinimide (**2**) through the formation of an ester bond in *N*-methoxypurpurinimide-18 (**3**), which prevents side processes involving the free OH group. The use of LiAlH_4_ in THF in the presence of two equivalents of AlCl_3_ ensures the reduction of both carbonyl groups of exocyclic ring E and the ester substituent at position 17^3^, yielding a new chlorin (**4**) containing a tetrahydropyridine fragment; its formation was confirmed by ^1^H NMR data, in particular by a broad multiplet in the region of 5.87–4.90 ppm, assigned to the protons at positions 13^1^ and 15^1^. The observed broadening of the ^1^H NMR signals of the reduced ring is attributed to the low energy barriers of large-amplitude motions—nitrogen inversion and ring pseudorotation—which cause rapid conformational exchange, dynamically averaging the proton environments and leading to a broadening of the resonance lines, especially for protons located near the nitrogen atom or in sterically sensitive positions [61].

### 3.2. Synthesis of the Thiocarbonyl Chlorin Derivative

The obtained data indicate that the thiolation of the methyl ester of *N*-methoxypurpurinimide (**3**) with Lawesson’s reagent proceeds selectively at the carbonyl group in position 13^1^ of exocycle E; this regioselectivity is explained by the greater steric accessibility of this carbonyl group compared to the other carbonyl functionality of the exocycle, which directs the attack of the thiolating agent specifically to position 13^1^, resulting in the replacement of the oxygen atom by sulfur to give the thioimide derivative (**5**) as a new representative of the purpurinimide class.

The structure of compound (**5**) is confirmed by the observed changes in the NMR spectra relative to the starting compound (**3**): in the ^1^H NMR spectrum, the downfield shift of the signal corresponding to the methyl protons at position 12 of the macrocycle reflects the altered electronic environment of the macrocyclic system upon the introduction of sulfur into the exocycle, while in the ^13^C NMR spectrum, the downfield shift of the signal assigned to C-13^1^ [62] is consistent with the replacement of oxygen by the less electronegative but more polarizable sulfur atom, which affects the chemical shift of the adjacent carbon; taken together, these spectral changes confirm the selective formation of the thioimide derivative (5) with the sulfur atom located in position 13^1^ of exocycle E.

### 3.3. Study of the Spectral and Photophysical Properties of the Synthesized Compounds

The introduction of a sulfur atom into the chromophore reduces the HOMO-LUMO energy gap, which increases spin–orbit coupling and facilitates intersystem crossing (ISC). These effects manifest in compound (**5**) as a bathochromic shift of the long-wavelength absorption and fluorescence maxima, fluorescence quenching and enhanced singlet-oxygen production. A similar correlation between thionation, enhanced ISC and reduced fluorescence quantum yield has been reported for a number of heavy-atom-free photosensitizers [51,68].

In the reduced derivative (**4**) the absorption and fluorescence bands undergo a hypsochromic shift owing to decreased conjugation between the chlorin macrocycle and the exocyclic E-ring, which increases the energy gap between the ground and first excited states. It is well established that the degree of reduction and the peripheral structure of chlorin macrocycles can significantly affect their electronic structure and photophysical properties [69,70].

Upon transfer from chloroform to aqueous medium, compounds (**3**)–(**5**) exhibit substantial changes in their fluorescence and singlet oxygen quantum yields, whereas the positions of their long-wavelength absorption and fluorescence maxima remain almost unchanged: the shifts do not exceed 4 nm. At the same time, the quantum yields vary markedly: φ*_Δ_* decreases by a factor of 1.5–6 for all compounds and φ*_fluo_* remains unchanged for derivative (**5**), while for compounds (**3**) and (**4**) it increases 1.3-fold and decreases 3.9-fold, respectively.

The photophysical properties of porphyrin-based photosensitizers are known to be solvent-dependent. For instance, changes in medium polarity can alter the fluorescence and singlet oxygen quantum yields by redistributing excited-state population among radiative, non-radiative, and intersystem crossing channels. For certain chlorins, this solvent dependence may also arise from aggregation of the fluorophore [71,72]. However, no spectrophotometrically detectable signs of aggregation were observed for compounds (**3**)–(**5**). Thus, the reduced photophysical performance in aqueous media is most likely attributable to alterations in the chromophore’s microenvironment and in the kinetics of excited-state relaxation.

For compound (**3**), the increase in φ*_fluo_* accompanied by a decrease in φ*_Δ_* suggests a shift in the balance between fluorescence and intersystem crossing: less efficient ISC results in a larger fraction of excited molecules relaxing radiatively and, consequently, a lower yield of triplet states responsible for ^1^O_2_ generation. In contrast, for compound (**4**), the simultaneous sharp drop in both parameters points to enhanced non-radiative deactivation of the excited state in an aqueous environment.

To exclude potential interference from other reactive oxygen species (ROS), we selected rubrene as a probe for ^1^O_2_ via [4 + 2]-cycloaddition. Specifically, rubrene reacts selectively with 1 ^1^O_2_ to form a stable endoperoxide adduct, resulting in pronounced bleaching of its characteristic absorption band. Under these conditions, rubrene remains chemically inert toward other ROS, including superoxide anion radicals and hydrogen peroxide [73]. Similarly, anthracene derivatives such as ABDA serve as highly specific ^1^O_2_ scavengers. Their bleaching occurs exclusively through ^1^O_2_ -mediated Diels–Alder endoperoxide formation and remains virtually unaffected by hydroxyl radicals within the specified working concentration ranges [74].

### 3.4. Quantum-Chemical Calculations of Energy Levels of Molecular Orbitals Using the DFT Method

**Figure 4:**
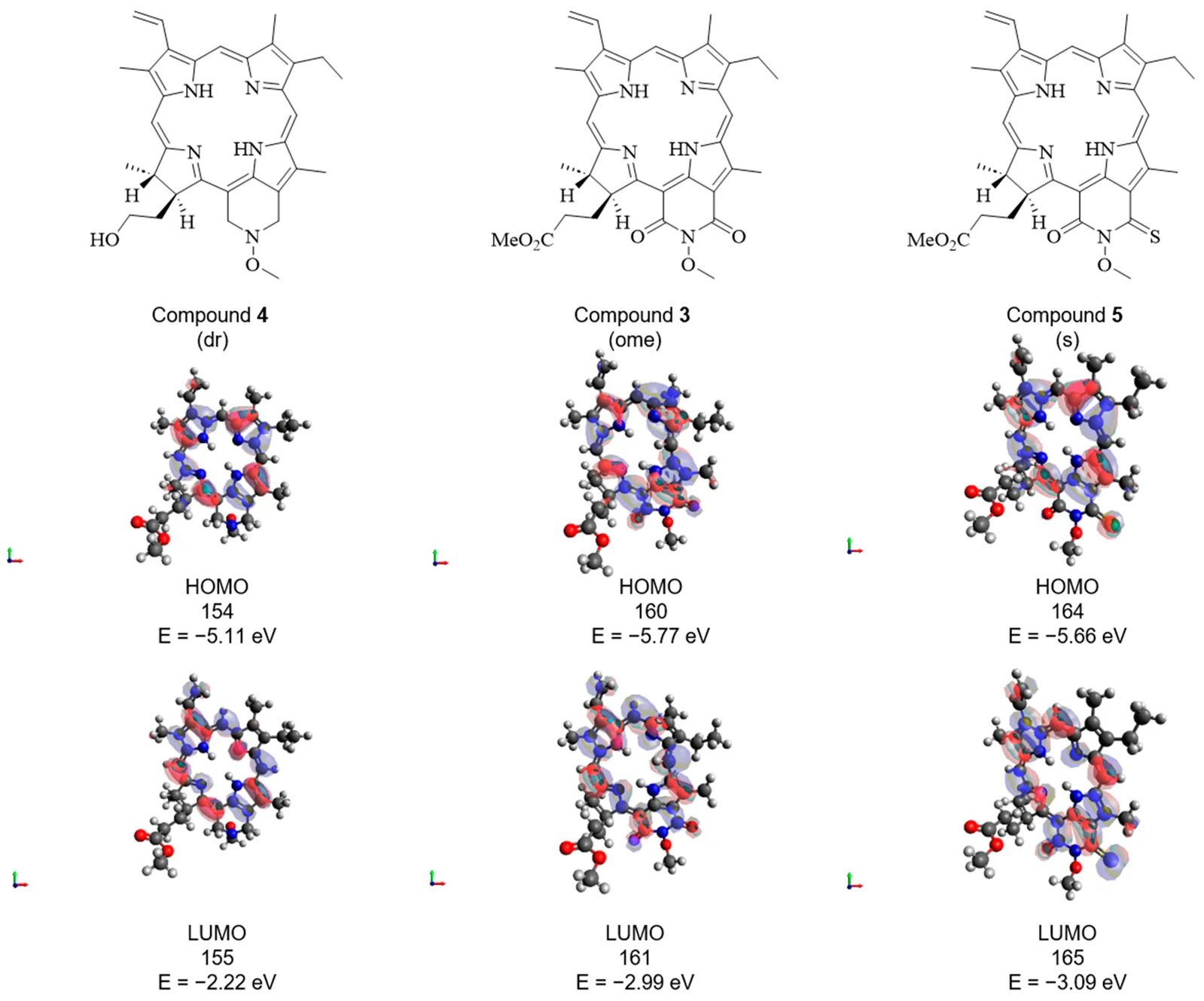
Visualization of the distribution of electron densities of HOMO and LUMO orbitals for compounds (**3**), (**4**) and (**5**); DEF2-TZVP and PW6B95.

The comparative Kohn–Sham MO energy levels diagram (Figure 2) and the visualization of the electron density distribution (Figure 4) provide valuable insight into the observed spectral shifts due to structural differences in the exocycle E.

The electronic structure of compound (**4**) bearing the tetrahydropyridine fragment is most consistent with Gouterman’s four-orbital model [75,76]. The four relevant frontier orbitals—HOMO − 1, HOMO, LUMO, and LUMO + 1—are energetically well separated from the remaining MOs.

For the starting compound (**3**), the energies of the HOMO- and LUMO-orbitals are reduced compared to compound (**4**). This decrease seems to be associated with a slightly greater delocalization of MOs on carbonyl groups in the cyclic imide fragment of *N*-methoxypurpurinimide (**3**) compared to chlorin (**4**). These orbital energy shifts lead to a significant reduction in the HOMO-LUMO energy gap, qualitatively explaining the observed bathochromic displacement of the *Q*-bands in the region of 708 nm.

When replacing the oxygen atom in compound (**4**) with sulfur in compound (**5**), the HOMO energy increased, whereas the LUMO energy decreased slightly, which may be attributed to the redistribution of electron density induced by the chalcogen atom (Figure 4). Concomitantly, the HOMO-LUMO energy gap decreased substantially, consistent with the observed bathochromic shift of the *Q*-band to approximately 741 nm.

### 3.5. Experimental Assessment of Photoinduced and Dark Bactericidal Activity of the Obtained Compounds In Vitro

Overall, these results indicate that derivatives (**4**) and (**5**) exhibit enhanced antibacterial activity at low concentrations, particularly under irradiation, highlighting their potential as more effective photosensitizers compared to the parent compound (**3**).

The observed antibacterial effects correlate well with the photophysical properties of the studied compounds, specifically with their ability to generate singlet oxygen (φ*_Δ_*). Under irradiation, the antibacterial activity is largely determined by the quantum yield of singlet oxygen generation, since ^1^O_2_ is the principal cytotoxic species responsible for photoinduced bacterial inactivation. Compound (**5**), which exhibited the highest φ*_Δ_* in dichloromethane (0.89), also showed the most pronounced photoinduced inhibition at low concentrations, consistent with its efficient intersystem crossing and enhanced ^1^O_2_ production. Compound (**4**), despite a lower φ*_Δ_* (0.44), displayed a 2–4-fold higher inhibition than the parent compound (**3**) upon irradiation at a concentration of 3.175 μM. This phenomenon may be attributed to enhanced bioavailability at the low concentration tested. Specifically, it may result from superior penetration through the bacterial cell envelope (wall or membrane) or a more favorable partition coefficient between the aqueous medium and the bacterial cells. Furthermore, the presence of the tetrahydropyridine moiety may facilitate alternative modes of interaction with cellular targets, such as membrane disruption or binding to intracellular components, which act synergistically with the photodynamic effect. At higher concentrations, the differences between compounds (**4**) and (**5**) and the parent compound (**3**) became less pronounced, probably because the overall ^1^O_2_ production becomes sufficient to reach a plateau in the antibacterial response.

Notably, the dark antibacterial activity of compounds (**4**) and (**5**) also exceeded that of compound (**3**) at low concentrations (1.5–2-fold higher for both strains). This dark toxicity is unrelated to singlet oxygen generation, since ^1^O_2_ production requires photoexcitation, and likely reflects additional mechanisms such as dark redox activity, interaction with bacterial membranes, or intrinsic toxicity of the modified exocyclic ring. Nevertheless, the most significant enhancement of antibacterial activity was observed under irradiation, supporting the conclusion that the photosensitizing pathway, mediated by singlet oxygen, plays a decisive role in the observed bactericidal effect.

Taken together, these findings demonstrate that the introduction of a tetrahydropyridine fragment (compound **4**) or a sulfur atom (compound **5**) into the exocyclic ring of the purpurinimide macrocycle enhances both photoinduced and dark antibacterial activity, with the photoinduced effect correlating with the efficiency of singlet oxygen generation.

## 4. Materials and Methods

### 4.1. Materials

The organic solvents used were prepared and purified according to standard methods. The course of the reaction was monitored using analytical thin-layer chromatography (TLC) on Kieselgel 60 F254 plates (Merck, Darmstadt, Germany). Kieselgel 60 (40/63 μm) (Merck, Darmstadt, Germany) silica gel was used for column chromatography and preparative TLC. Spectra of nuclear magnetic resonance (NMR) were recorded on the Bruker DPX-300 spectrometer (Bruker, Alzenau, Germany) with CDCl_3_ as a solvent. ESI-MS mass spectra were obtained on the Bruker MicrOTOF II mass spectrometer (Bremen, Germany) using the method of electrospray ionization (ESI). Electronic absorption and fluorescence spectra were recorded in CH_2_Cl_2_ and in 4% micellar solution of Kolliphor^®^ ELP on the Shimadzu UV-1800 (Shimadzu, Duisburg, Germany) spectrophotometer and the fluorat-02-panorama (Lumex, Sant-Peterburg, Russia), respectively. Chlorine *E*_6_ [77,78] was used as a standard for measuring the quantum yield of fluorescence in dichloromethane and a 4% micellar solution of Kolliphor^®^ ELP as well as singlet oxygen generation in a 4% micellar solution of Kolliphor^®^ ELP. To measure the quantum yield of singlet oxygen generation in dichloromethane, methylene blue was used as a standard [79,80]. To measure the quantum yield of singlet oxygen generation, 5,6,11,12-tetraphenyltetracene (rubrene) [81,82] was used as a trap in dichloromethane and 2,2′-(anthracene-9,10-diylbis(methylene))dimalonic acid [83] in a 4% micellar solution of Kolliphor^®^ ELP, the light source was a red LED (λ = 680 ± 20 nm, Ps = 16 MW/cm^2^; λ = 740 ± 20 nm, Ps = 10 MW/cm^2^). The bacterial strains were cultured in Mueller–Hinton broth. To study photoinduced cytotoxicity, an LED-based irradiator was used (λ = 660 ± 10 nm, Ps = 7 MW/cm^2^) as a light source. An iMark photometer (Bio-Rad, Hercules, CA, USA) was used to measure the optical density of bacterial suspensions. A Biosan microspin 12 (Biosan, Riga, Latvia) centrifuge was used to separate cell cultures from a liquid medium.

### 4.2. Synthesis

Purpurin-18 (**1**), *N*-hydroxypurpurinimide (**2**) and its methylated derivative (**3**) was obtained using methods known in the scientific literature [57,58,59].

#### 4.2.1. 17^3^-Hydroxy-13^1^,13^1^,15^1^,15^1^-tetrahydro-*N*-methoxypurpurinimide (**4**)

*N*-methoxypurpurinimide (**3**) (30 mg, 0.05 mmol) was dissolved in tetrahydrofuran (THF) (25 mL) and lithium aluminum hydride (LiAlH_4_) (10 mg, 0.26 mol) and 2 equivalents of aluminum chloride (AlCl_3_) (13.6 mg, 0.1 mmol) were added at 0 °C. The mixture was allowed to warm to room temperature with stirring in the dark. After 24 h, 10 mL of dichloromethane and 1 mL of 1M HCl solution were added to the reaction mass and stirred for another 1 h. After that, extraction was carried out, the organic layer was passed through Na_2_SO_4_ and concentrated under pressure on a rotary evaporator. The resulting product was purified by column chromatography, eluting with a gradient from pure dichloromethane to a 1:10 (*v*/*v*) mixture of methanol and dichloromethane. The reaction yield was 45% (13.5 mg). UV/VIS (CH_2_Cl_2_) λ_max_, nm (ε, M^−1^ × cm^−1^): 400 (150,080), 500 (22,160), 600 (62,400), 656 (64,280). ESI *m*/*z* found [M + H]^+^ 552.30, calculated 552.33. ^1^H NMR (300 MHz, CDCl_3_) δ 9.85 (1H, s, 10-H), 9.72 (1H, s, 5-H), 8.95 (1H, s, 20-H), 8.22 (1H, dd, *J* = 17.9, 11.4 Hz, 3^1^-CH), 6.38 (1H, d, *J* = 17.9 Hz, 3^2^-CH_2_), 6.17 (1H, d, *J* = 10.5 Hz, 3^2^-CH_2_), 5.87–4.90 (4H, m, 13^1^,13^2^-CH_2_), 4.69 (1H, s, 17^3^-OH), 4.66–4.54 (2H, m, 17-H, 18-H), 4.02 (3H, s, 13^4^-CH_3_), 3.88 (2H, q, *J* = 7.5 Hz, 8^1^-CH_2_), 3.58 (3H, s, 12-CH_3_), 3.55 (3H, s, 2-CH_3_), 3.42 (3H, s, 7-CH_3_), 2.43–2.10 (2H, m, 17^3^-CH_2_), 2.03–1.88 (2H, m, 17^1^-CH_2_), 1.85 (3H, d, *J* = 7.4 Hz, 18-CH_3_), 1.78 (3H, t, *J* = 7.6 Hz, 8^2^-CH_3_), 1.64–1.43 (2H, m, 17^2^-CH_2_), −2.41 (1H, s, NH), −2.59 (1H, s, NH). ^13^C NMR (75 MHz, CDCl_3_) δ 167.95, 165.30, 164.34, 151.75, 149.95, 143.60, 137.58, 136.29, 133.88, 133.37, 132.79, 129.95, 129.73, 128.78, 126.59, 121.17, 102.95, 99.26, 98.79, 93.48, 62.71, 60.25, 58.11, 54.17, 53.18, 49.12, 31.41, 29.38, 24.11, 19.78, 17.80, 12.34, 11.51, 11.26. Elemental analysis for CHN, calculated for C_34_H_41_N_5_O_2_ (%): C, 74.02; H, 7.49; N, 12.69. Found (%): C, 74.04; H, 7.50; N, 12.68.

#### 4.2.2. Methyl Ester of *N*-Methoxypurpurintioimide (**5**)

*N*-methoxypurpurintioimide (**3**) (50 mg, 0.08 mmol) was dissolved in toluene (2 mL) and 2 equivalents of the Lawesson’s reagent (68.4 mg, 0.16 mmol) were added. The reaction mixture was stirred at 110 °C under reflux for 6 h in an argon atmosphere. The resulting compound was purified by preparative thin-layer chromatography using a methanol/dichloromethane solvent system (1:400, *v*/*v*). The yield of *N*-methoxypurpurinthioimide methyl ester (**5**) was 30% (15.1 mg). UV/VIS (CH_2_Cl_2_) λ_max_, nm (ε, M^−1^ × cm^−1^): 370 (59,160), 440 (74,080), 572 (41,500), 686 (14,980), 742 (53,040). ESI *m*/*z* found [M + H]^+^ 624.32, calculated 624.26. ^1^H NMR (300 MHz, CDCl_3_) δ 9.45 (1H, s, 10-H), 9.17 (1H, s, 5-H), 8.46 (1H, s, 20-H), 7.81 (1H, dd, *J* = 17.8, 11.7 Hz, 3^1^-CH), 6.26 (1H, d, *J* = 17.7 Hz, 3^2^-CH_2_), 6.15 (1H, d, *J* = 11.7 Hz, 3^2^-CH_2_), 5.27 (1H, d, *J* = 9.1 Hz, 17-H), 4.42 (3H, s, 13^4^-CH_3_), 4.31 (1H, q, *J* = 13.9, 7.0 Hz, 18-H), 3.78 (3H, s, 12-CH_3_), 3.61 (3H, s, 17^4^-CH_3_), 3.51 (2H, q, *J* = 15.0, 7.4 Hz, 8^1^-CH^2^), 3.30 (3H, s, 2-CH_3_), 3.06 (3H, s, 7-CH_3_), 2.88–2.77 (1H, m, 17^1^-CH_2_), 2.56–2.46 (2H, m, 17^1^-CH_2,_ 17^2^-CH_2_), 2.03–1.94 (1H, m, 17^2^-CH_2_), 1.75 (2H, d, *J* = 7.2 Hz, 18-CH_3_), 1.61 (3H, t, *J* = 7.5 Hz, 8^2^-CH_3_), 0.58 (1H, s, NH), 0.1 (1H, s, NH). ^13^C NMR (75 MHz, CDCl_3_) δ 184.19, 177.83, 175.51, 173.80, 161.07, 156.13, 150.04, 145.77, 143.89, 142.20, 137.39, 136.41, 136.30, 135.24, 132.37, 131.27, 128.41, 123.32, 120.70, 107.86, 103.56, 97.09, 95.27, 63.54, 55.40, 51.57, 48.92, 32.64, 31.15, 23.86, 19.27, 17.36, 14.95, 11.86, 10.93. Elemental analysis for CHNS, calculated for C_35_H_37_N_5_O_4_S (%): C, 67.39; H, 5.98; N, 11.23; S, 5.14. Found (%): C, 67.40; H, 6.00; N, 11.21; S, 5.13.

### 4.3. Calculation of the Fluorescence Quantum Yield

The fluorescence emission spectra were recorded with a wavelength step of 1 nm, and in the range of 600–800 nm, with an excitation wavelength of 530 nm. The sensitivity of the photomultiplier was adjusted to ensure a high signal-to-noise ratio without saturating the detector.

To measure fluorescence, test sample solutions (2 mL) were prepared with an optical density of ~0.1 at the long-wavelength absorption maximum, both in dichloromethane and in a 4% micellar solution of Kolliphor^®^ ELP. Chlorine *E*_6_ [77,78] was used as a standard for measuring the quantum yield of fluorescence in dichloromethane and a 4% micellar solution of Kolliphor^®^ ELP. Fluorescence quantum yields were calculated by a relative method [66], taking into account the integrated peak area and the peak intensity.

### 4.4. Calculation of the Quantum Yield of Singlet Oxygen Generation

To measure the singlet oxygen quantum yield in dichloromethane solutions (2 mL), the test samples and the standard (methylene blue [79,80]) were prepared with an absorbance of ~0.2 A. Rubrene [81,82] trap solution was also prepared with an absorbance 10 times higher than that of the test compounds. For the measurement, 0.5 mL of the compound solution and 0.5 mL of the trap solution were added to the quartz cuvette (optical path length 1 cm). Absorbance was recorded at four time points (0, 10, 20, and 40 s).

For measurements in a 4% micellar solution of Kolliphor^®^ ELP solution, the test samples and the standard (chlorin *E*_6_ [77,78]) were prepared analogously. The trap solution (2,2′-(anthracene-9,10-diylbis(methylene))dimalonic acid [83]) had a concentration of 10 × 10^−5^ mol/L (A = 0.897). For each measurement, 0.5 mL of the sample and 0.5 mL of the trap were mixed in the quartz cuvette (optical path length 1 cm). Absorbance was recorded at seven time points (0, 10, 20, 30, 50, 90, and 150 s).

All solutions were prepared and measurements were performed in the dark. The generation of singlet oxygen was monitored upon irradiation of the compounds under study at their respective long-wavelength absorption maxima. The quantum yield was determined mathematically using a modified version of a previously reported method [68,69], taking into account the change in the trap’s maximum absorbance between the initial and final time points, as well as the wavelength and peak intensity.

### 4.5. Computational Details

To optimize the geometry, calculate the energies of the MOs and obtain cube files to visualize the distribution of the electron density of the HOMO and LUMO orbitals for compounds (**3**)–(**5**). The base/functional combination def2-TZVP/PW6B95 [66,67], implemented in the Orca 6.0.1 [84,85,86] software, was used. The HOMO and LUMO electron-density distributions were visualized using Avogadro software 1.2.0.

### 4.6. Studies of Biological Antibacterial Activity

Micellar solutions of the analyzed compounds were prepared according to a standard technique described earlier by our scientific group [55]. To evaluate antibacterial activity, reference strains of Gram-positive *Staphylococcus aureus* ATCC 25923 and Gram-negative bacterium *Pseudomonas aeruginosa* ATCC 27853 were used. A total of 600 μL of microbial suspensions in a concentration of 109 CFU/mL were selected and placed in centrifugal test tubes, then micellar solutions of the analyzed compounds were placed in them until the final concentrations of the photosensitizer were reached—3.125, 6.25, 12.5, 25 and 50 μM, respectively—after which the cells were incubated for 30 min. Next, the resulting suspensions were centrifuged and the additive liquid was removed. The precipitate was re-suspended in 600 μL of phosphate-salt buffer (PBS). The centrifugation and flushing procedures were repeated twice. Finally, the resulting sediment was resuspended in 600 μL of Mueller–Hinton broth.

We took 100 μL of the resulting suspensions and transferred them to a 96-well microplate. Then one set of wells was irradiated with red light (Ps = 10 mW/cm^2^) to achieve a light dose of 10 J/cm^2^; the other set was kept in the dark. For each compound, the light dose was taken into account by adjusting the irradiation time according to its absorption spectrum. The viability of microorganisms was determined photometrically by measuring the optical density in each well after incubation of microtablets in the dark at 37 °C for 24 h.

As a positive control, test strains were used that were exposed to identical conditions, including light irradiation, but without incubation with photosensitizers. A negative control was presented by test strains that were neither exposed to light irradiation nor incubation with photosensitizers. All experimental procedures were performed in three repetitions.

Differences in biological activity among compounds (**3**)–(**5**) were assessed using a one-way analysis of variance (one-way ANOVA) followed by Tukey’s multiple comparison test. The data presented in the updated Table S4 are expressed as the mean ± confidence interval. To increase the statistical power of the analysis, the sample size for all studied compounds was increased (to *n* = 6). Corresponding changes have been made to Table S4 and Figure 3. In the figures, asterisks (*) indicate statistically significant differences in uptake (*p* < 0.05).

## 5. Conclusions

In this paper, for the first time, by reducing previously obtained *N*-methoxypurpurinimide (**3**) with lithium aluminum hydride in the presence of aluminum chloride, a chlorin containing a tetrahydropyridine fragment (**4**) was obtained. In addition, a method of obtaining a thioimide derivative (**5**) using a Lawesson’s reagent was proposed. For the latter, pronounced bathochromic shifts of the long-wavelength absorption and fluorescence maxima to 741 and 755 nm, respectively, were observed. These wavelengths fall within the spectral region characteristic of bacteriochlorins, indicating a close structural and electronic relationship between dihydro- and tetrahydroporphyrins. Using quantum chemical calculations, the molecular orbital energies were determined and subsequently used to construct a Kohn–Sham MO energy-level diagram for compounds (**3**)–(**5**). Changes in HOMO-LUMO MO energy values are qualitatively consistent with the positions of long-wave absorption maxima for chlorin derivatives (**4**)–(**5**). At a concentration of 3.175 uM, compounds (**4**) and (**5**) showed greater activity compared to the original chlorine (**3**) against both Gram-positive and Gram-negative strains: inhibition in the above-mentioned derivatives is 4.09 and 2.85 times higher, respectively, compared to *N*-methoxypurpurinimide (**3**).

Overall, the obtained results demonstrate that structural modification of the parent compound (**3**) can enhance its antibacterial activity, particularly at low concentrations under irradiation.

## Figures and Tables

**Table 1:** Spectral and photophysical properties of novel chlorin derivatives.

Compound/ Parameter	λ*_abs max_*, nm ^1^	λ*_abs max_*, nm ^2^	λ*_fluo max_*, nm ^1^	λ*_fluo max_*, nm ^2^	φ*_fluo_* ^1^	φ*_fluo_* ^2^	φ*_Δ_* ^1^	φ*_Δ_* ^2^
(**3**)	708	711	718	722	0.049	0.065	0.81	0.19
(**4**)	656	658	662	662	0.250	0.064	0.44	0.07
(**5**)	741	741	755	751	0.009	0.010	0.89	0.59

λ*_abs max_*—absorption maximum wavelength, λ*_fluo max_*—wavelength of maximum fluorescence emission, φ*_fluo_*—fluorescence quantum yield, φ*_Δ_*—quantum yield of singlet oxygen generation; ^1^—the measurements were taken in dichloromethane; ^2^—the measurements were taken in a 4% micellar solution of Kolliphor^®^ ELP.

## Data Availability

The original contributions presented in this study are included in the article/Supplementary Materials. Further inquiries can be directed to the corresponding authors.

## Supplementary Materials

The following supporting information can be downloaded at: https://www.mdpi.com/article/10.3390/ijms27219845/s1.

## Abbreviations

The following abbreviations are used in this manuscript:

AlCl_3_	aluminum chloride
aPDT	Antimicrobial photodynamic therapy
DFT	Density functional theory
DLS	Dynamic light scattering
ESI	Electrospray ionization
ISC	Intersystem crossing
LiAlH_4_	Lithium aluminum hydride
MO	Molecular orbital
NIR	Near-infrared region
NMR	Nuclear magnetic resonance
PBS	Phosphate-salt buffer
PDT	Photodynamic therapy
PS	Photosensitizers
ROS	Reactive oxygen species
TD-DFT	Time-dependent density functional theory
THF	Tetrahydrofuran
TLC	Thin-layer chromatography
