# Supplementary material for: Novel Chlorin Derivatives with a Modified Exocycle E: A Study of Spectral and Biological Properties
Source: Int J Mol Sci. 2026 Nov 3;27(21):9845. doi: 10.3390/ijms27219845 (PMC13763868; doi:10.3390/ijms27219845)
Supplement: Supplementary file 1 [file ijms-27-09845-s001.zip › ijms-4636125-supplementary.pdf]

Novel Chlorin Derivatives with a Modified Exocycle E: A Study of Spectral and Biological Properties

Viktor A. Pogorilyy [1,*], Julia S. Kartava [1,*], Tanaka F. Tandare [1], Victoria V. Shchelkova [1,2], Nikita V. Suvorov [1], Yuriy L. Vasil'ev [1,3], Sergey S. Dydykin [3], Ivan Varga [3,4] and Michael A. Grin [1]

[1] Institute of Fine Chemical Technologies, MIREA-Russian Technological University, 86 Vernadsky Avenue, Moscow 119571, Russia; takerroy@yandex.ru (T.F.T.); victoria.shchelkova@yandex.ru (V.V.S.); suvorov.nv@gmail.com (N.V.S.); y_vasiliev@list.ru (Y.L.V.); michael_grin@mail.ru (M.A.G.)

[2] Russian State University named after A. N. Kosygin (Technologies. Design. Art), str Malaya Kaluzhskaya 1, Moscow 115035, Russia

[3] Department of Operative Surgery and Topographic Anatomy, I.M. Sechenov First Moscow State Medical University (Sechenov University), str Trubetskaya 8\2, Moscow 119435, Russia;
dydykin_s_s@staff.sechenov.ru (S.S.D.); ivan.varga@fmed.uniba.sk (I.V.)

[4] Institute of Histology and Embryology, Faculty of Medicine, Comenius University in Bratislava,
Sasinkova 4, 811 08 Bratislava, Slovakia

* Correspondence: pogorilviktor@gmail.com (V.A.P.); kartava02@mail.ru (J.S.K.)

Contents:

Figure S1: ESI mass spectrum of compound (**4**).

Figure S2: ^{1}H NMR spectrum of compound (**4**).

Figure S3: ^{13}C NMR spectrum of compound (**4**).

Figure S4: ESI mass spectrum of compound (**5**).

Figure S5: ^{1}H NMR spectrum of compound (**5**).

Figure S6: ^{13}C NMR spectrum of compound (**5**).

Figure S7: (**a**) the electronic absorption spectra for compound (**3**)–(**5**) in a water solution of 4% Kolliphor® ELP (**b**) the electronic fluorescence spectra for the compound (**3**)–(**5**) in a water solution of 4% Kolliphor® ELP.

Figure S8: The electronic absorption spectra of compounds (**3**) (**a**), (**4**) (**b**) and (**5**) (**c**) and rubrene as a trap for measuring formation of singlet oxygen in dichloromethane.

Figure S9: The electronic absorption spectra of compounds (**3**) (**a**), (**4**) (**b**) and (**5**) (**c**) and 2,2'-(anthracene-9,10-diylbis(methylene))dimalonic acid as a trap for measuring formation of singlet oxygen in a water solution of 4% Kolliphor® ELP.

Figure S10: The electronic absorption spectra of compounds (**3**) (**a**), (**4**) (**b**) and (**5**) (**c**) at different concentrations in a water solution of 4% Kolliphor® ELP.

Figure S11: Particle size distribution by intensity using the DLS method for compounds (**3**) (**a**), (**4**) (**b**) and (**5**) (**c**) in a water solution of 4% Kolliphor® ELP.

Table S1: Shapes of molecular orbitals potentially involved in electronic transitions for compound (**4**).

Table S2: Shapes of molecular orbitals potentially involved in electronic transitions for compound (**3**).

Table S3: Shapes of molecular orbitals potentially involved in electronic transitions for compound (**5**).

Table S4: Dependence of the percentage of inhibition of bacterial lines by the studied compounds (**3**)-(**5**) on the concentration with and without irradiation.

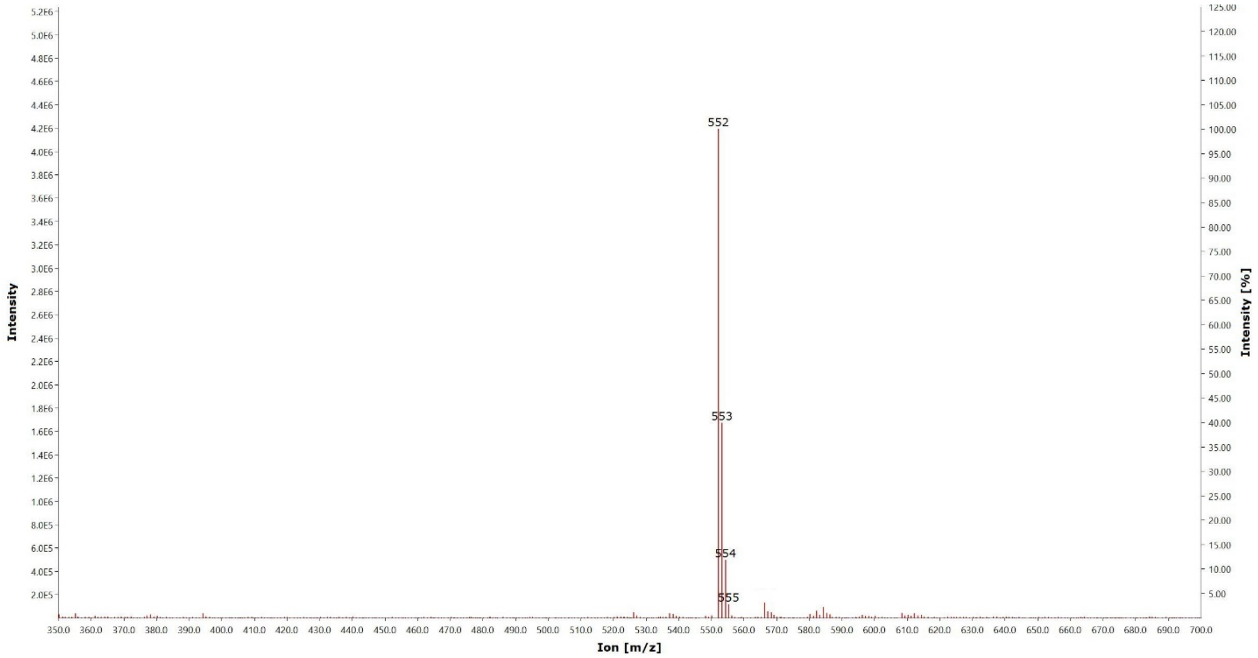

Figure S1: ESI mass spectrum of compound (**4**).

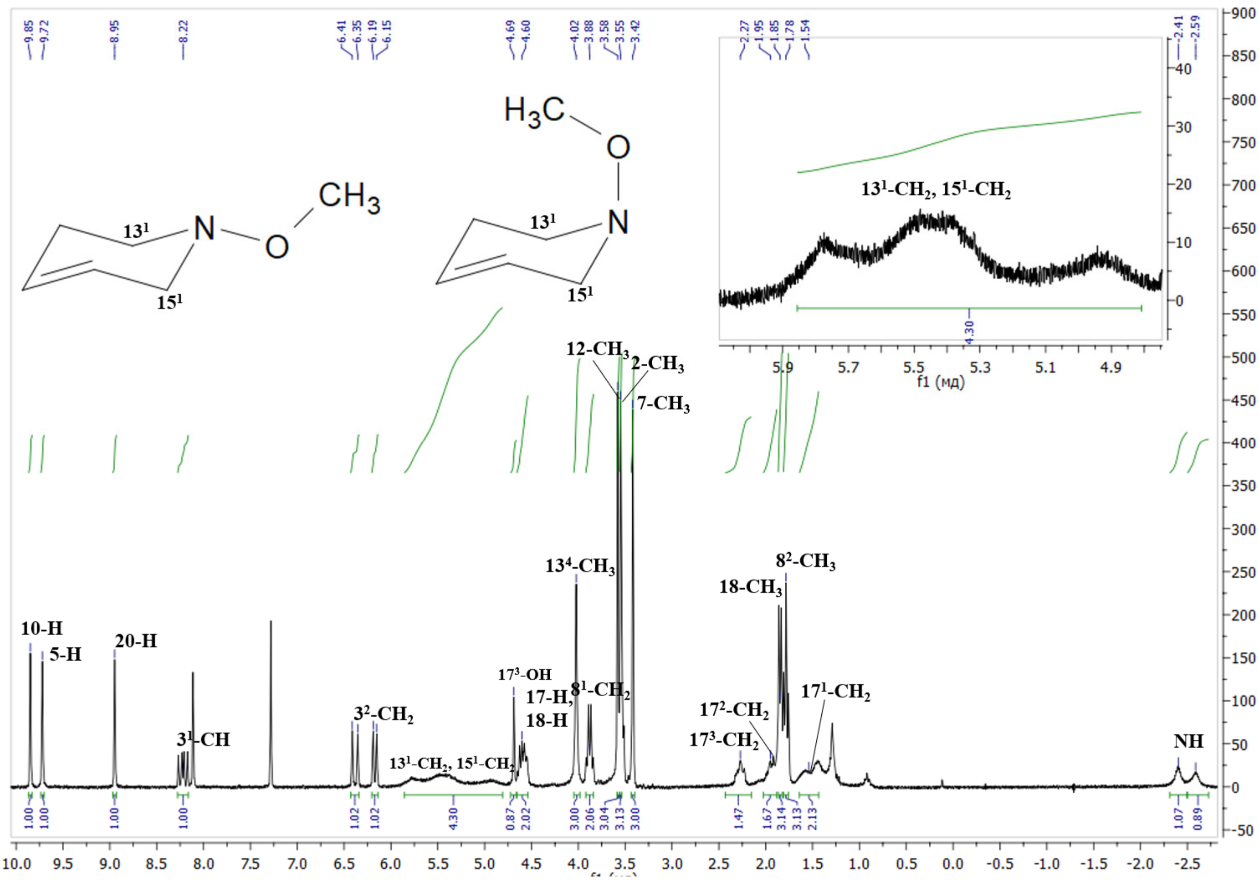

Figure S2: ^{1}H NMR spectrum of compound (**4**).

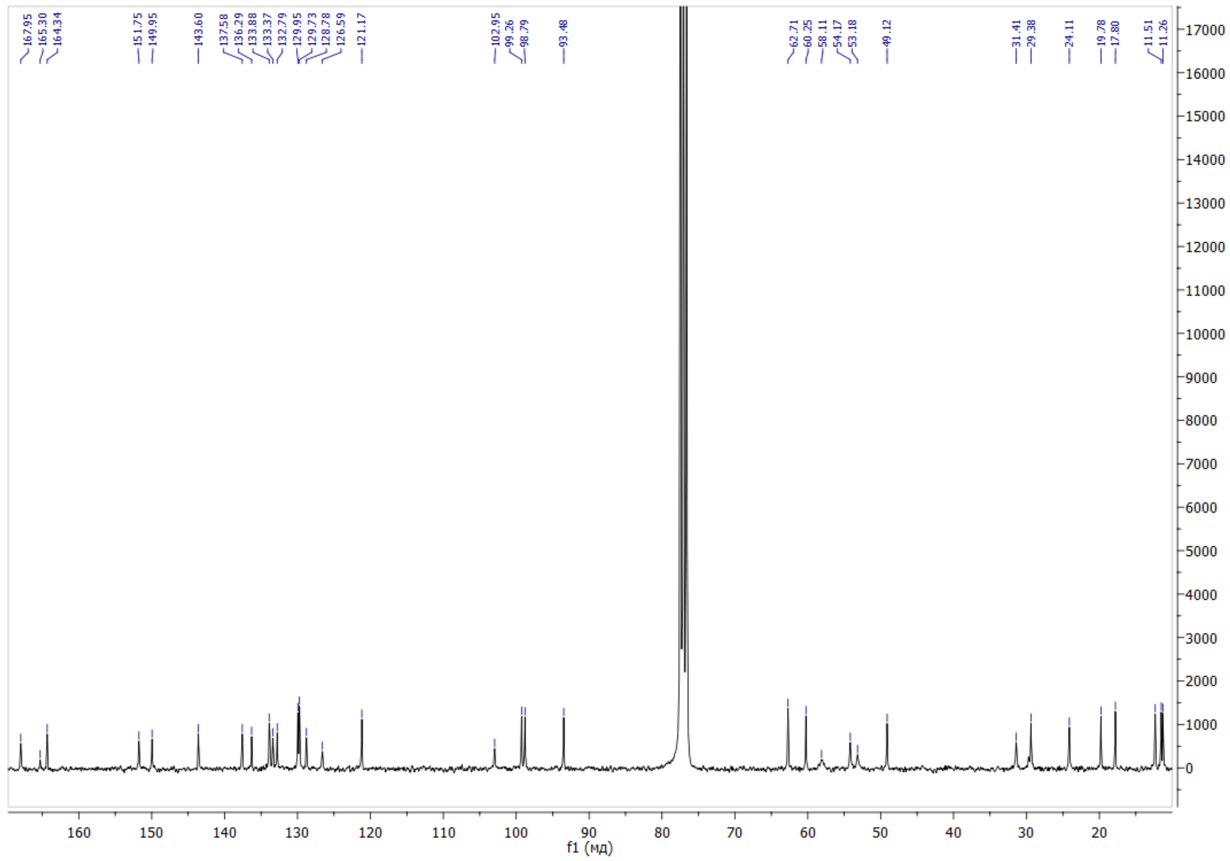

Figure S3: ^{13}C NMR spectrum 13C of compound (**4**).

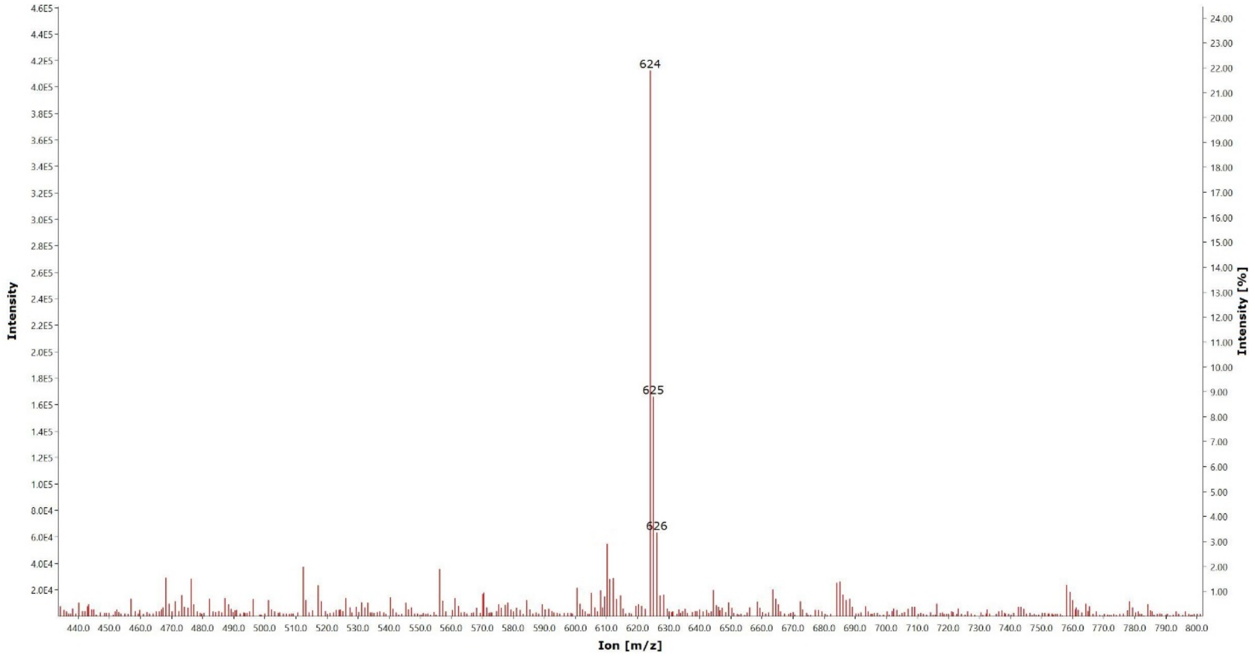

Figure S4: ESI mass spectrum of compound (**5**).

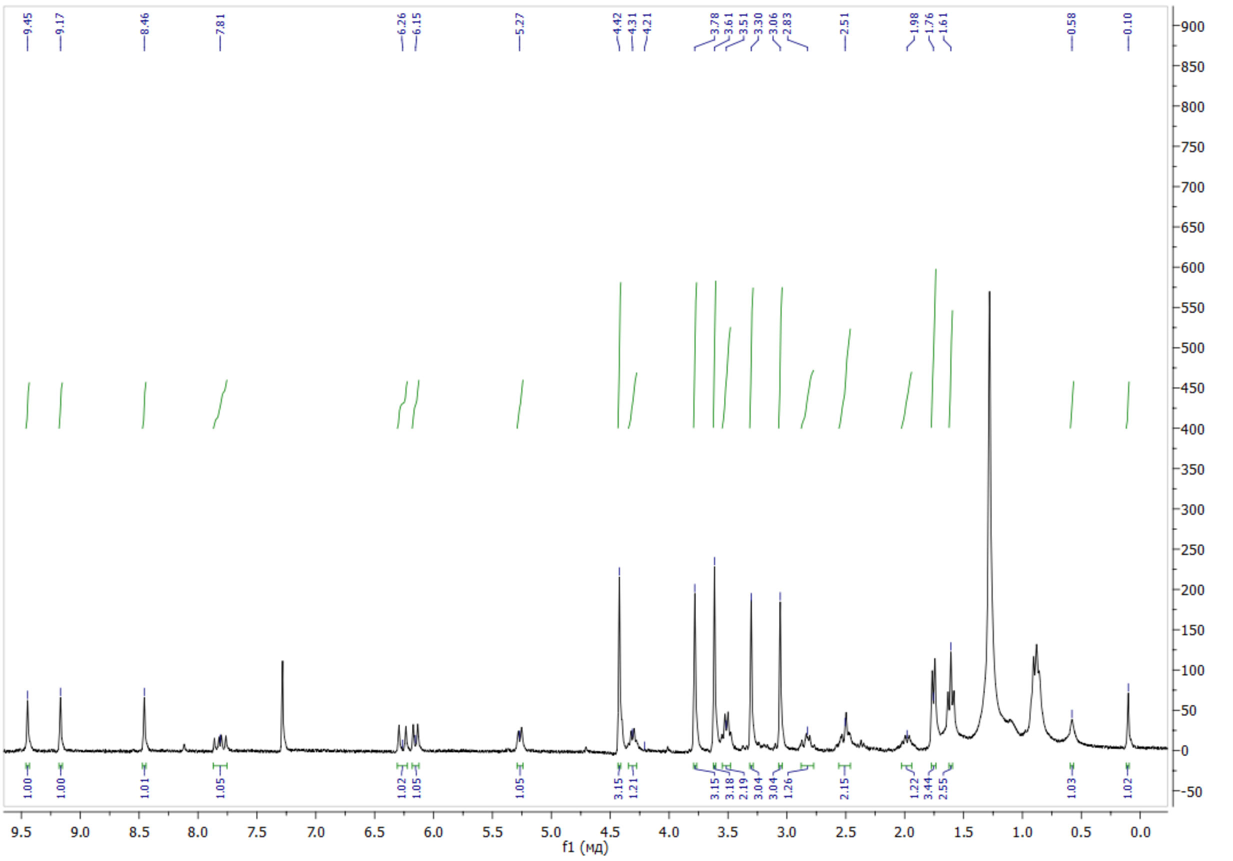

Figure S5: ^{1}H NMR spectrum of compound (**5**).

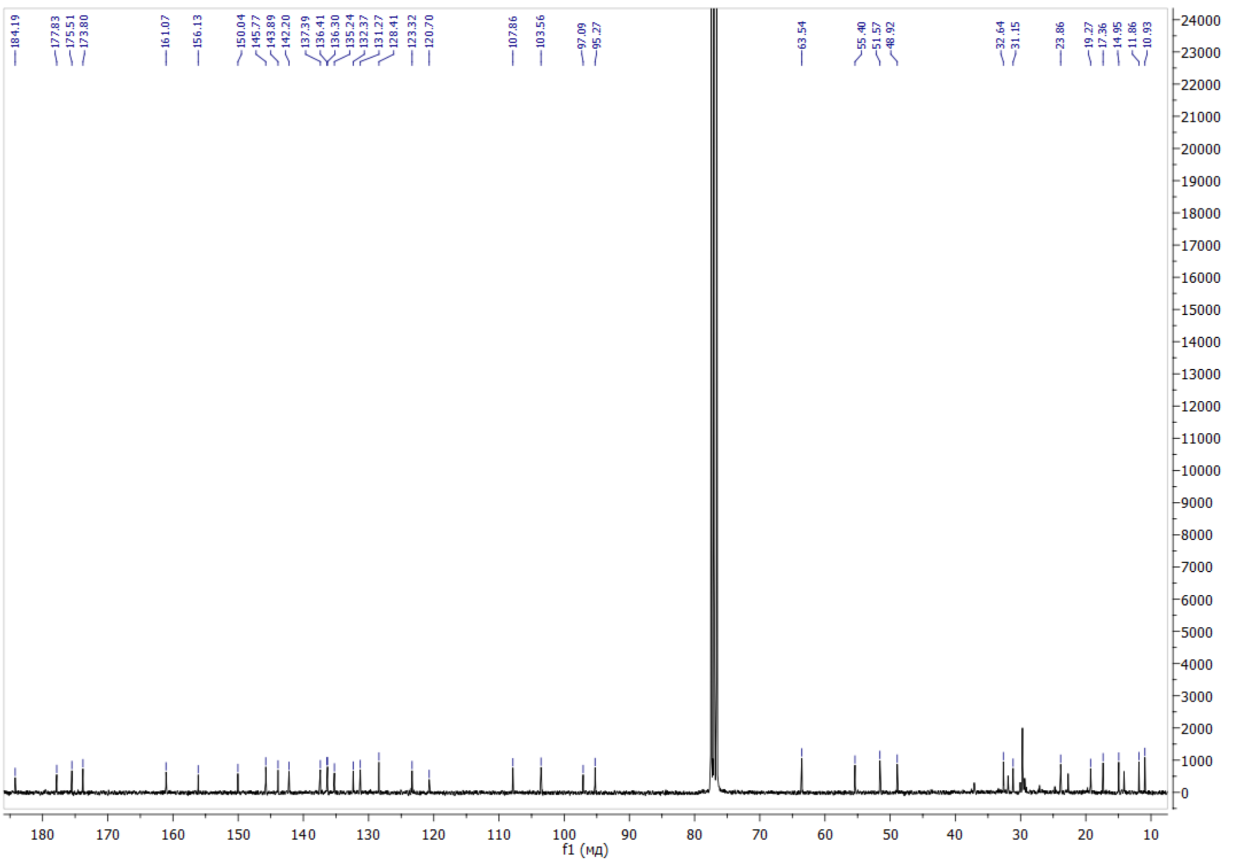

Figure S6: ^{13}C NMR spectrum of compound (**5**).

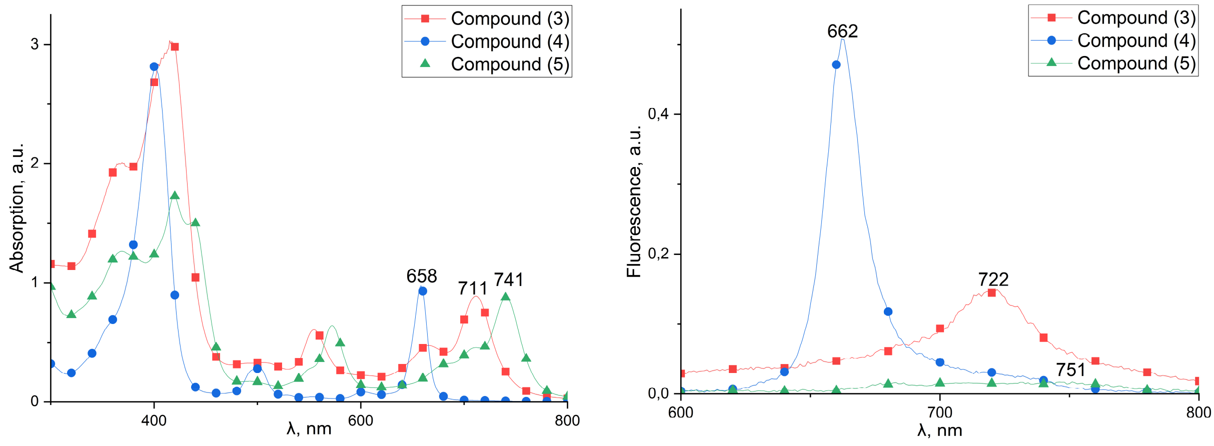

(**a**) (**b**)

Figure S7: (**a**) the electronic absorption spectra for compound (**3**)–(**5**) in a water solution of 4% Kolliphor® ELP (**b**) the electronic fluorescence spectra for the compound (**3**)–(**5**) in a water solution of 4% Kolliphor® ELP.

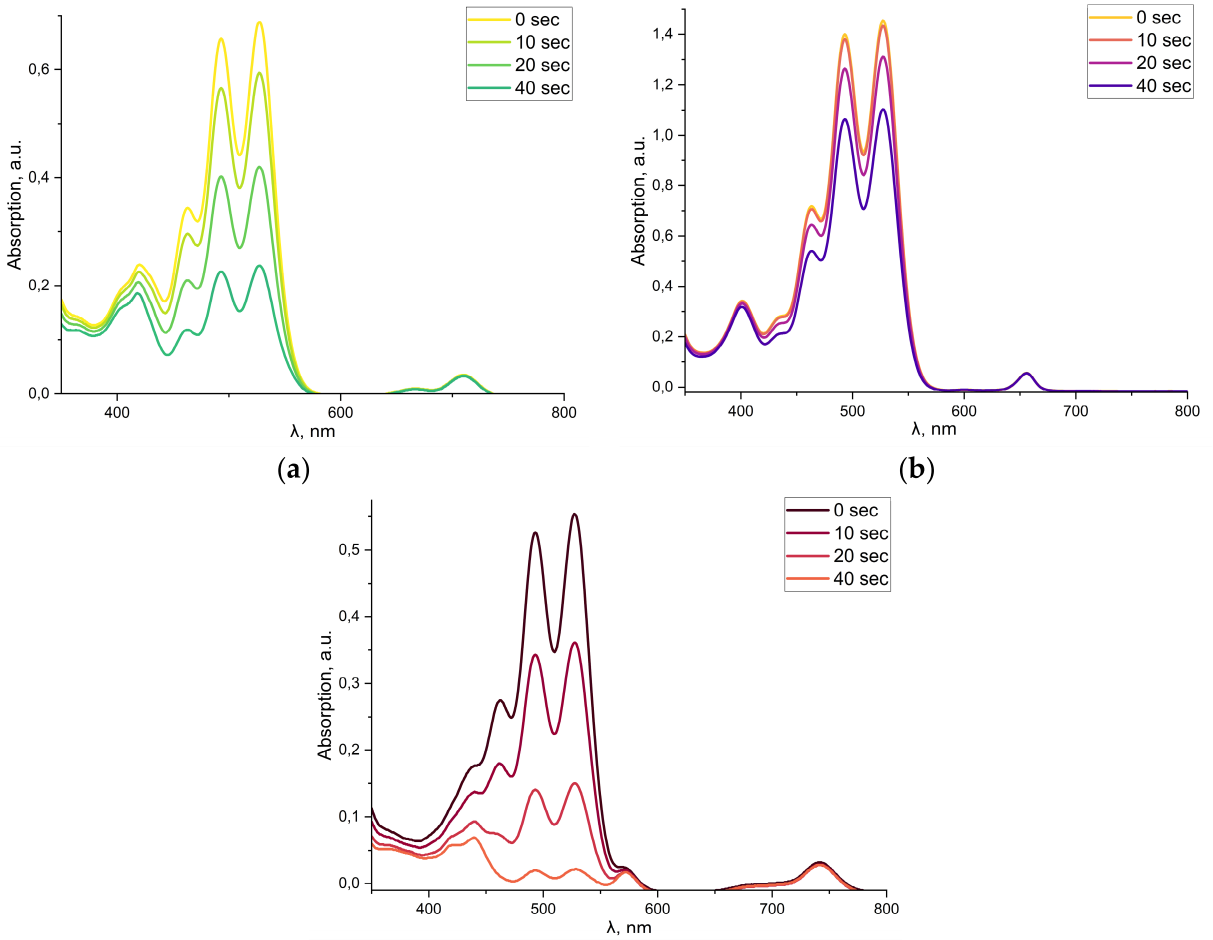

(**c**)

Figure S8: The electronic absorption spectra of compounds (**3**) (**a**), (**4**) (**b**) and (**5**) (**c**) and rubrene as a trap for measuring formation of singlet oxygen in dichloromethane.

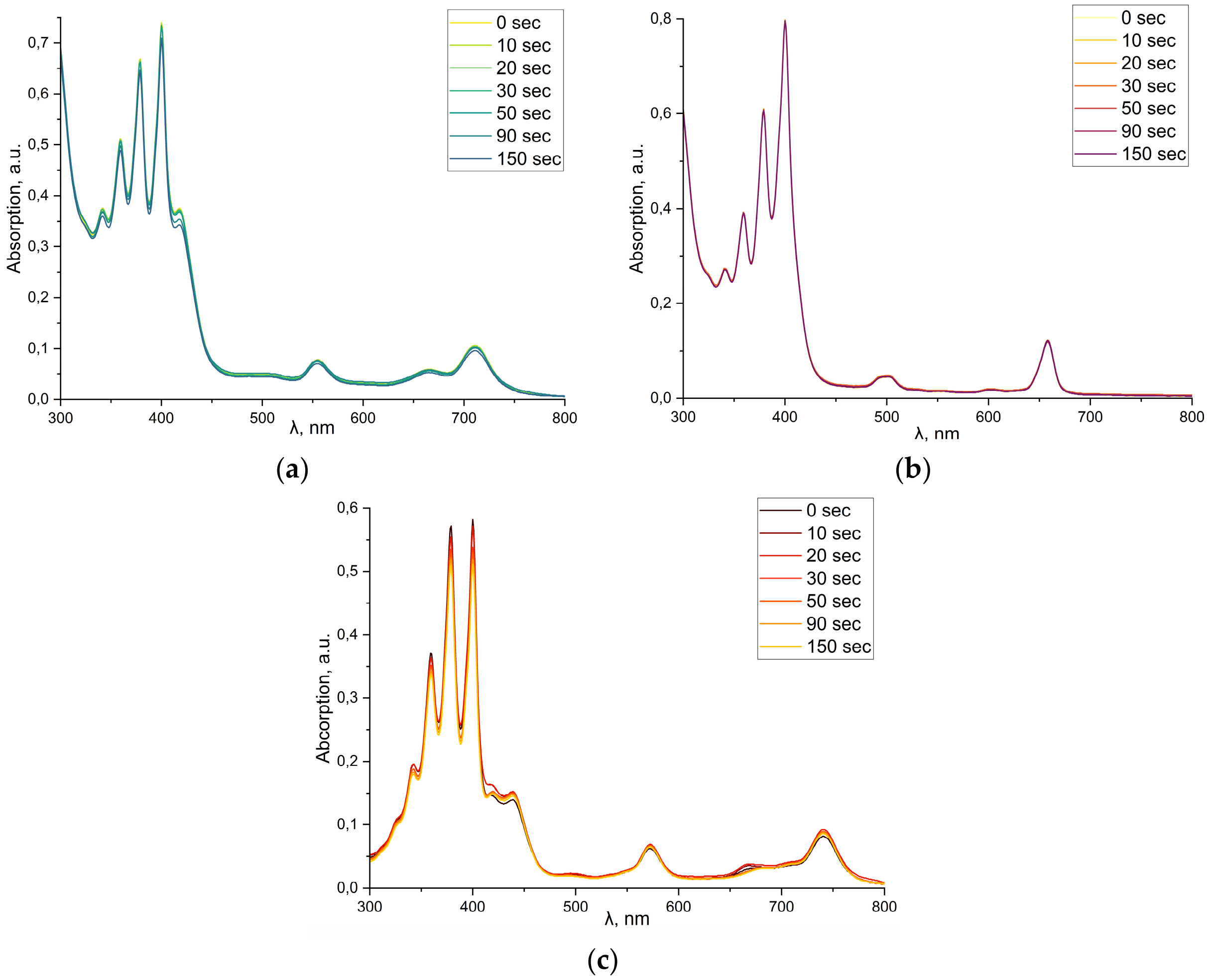

Figure S9: The electronic absorption spectra of compounds (**3**) (**a**), (**4**) (**b**) and (**5**) (**c**) and 2,2'-(anthracene-9,10-diylbis(methylene))dimalonic acid as a trap for measuring formation of singlet oxygen in a water solution of 4% Kolliphor® ELP.

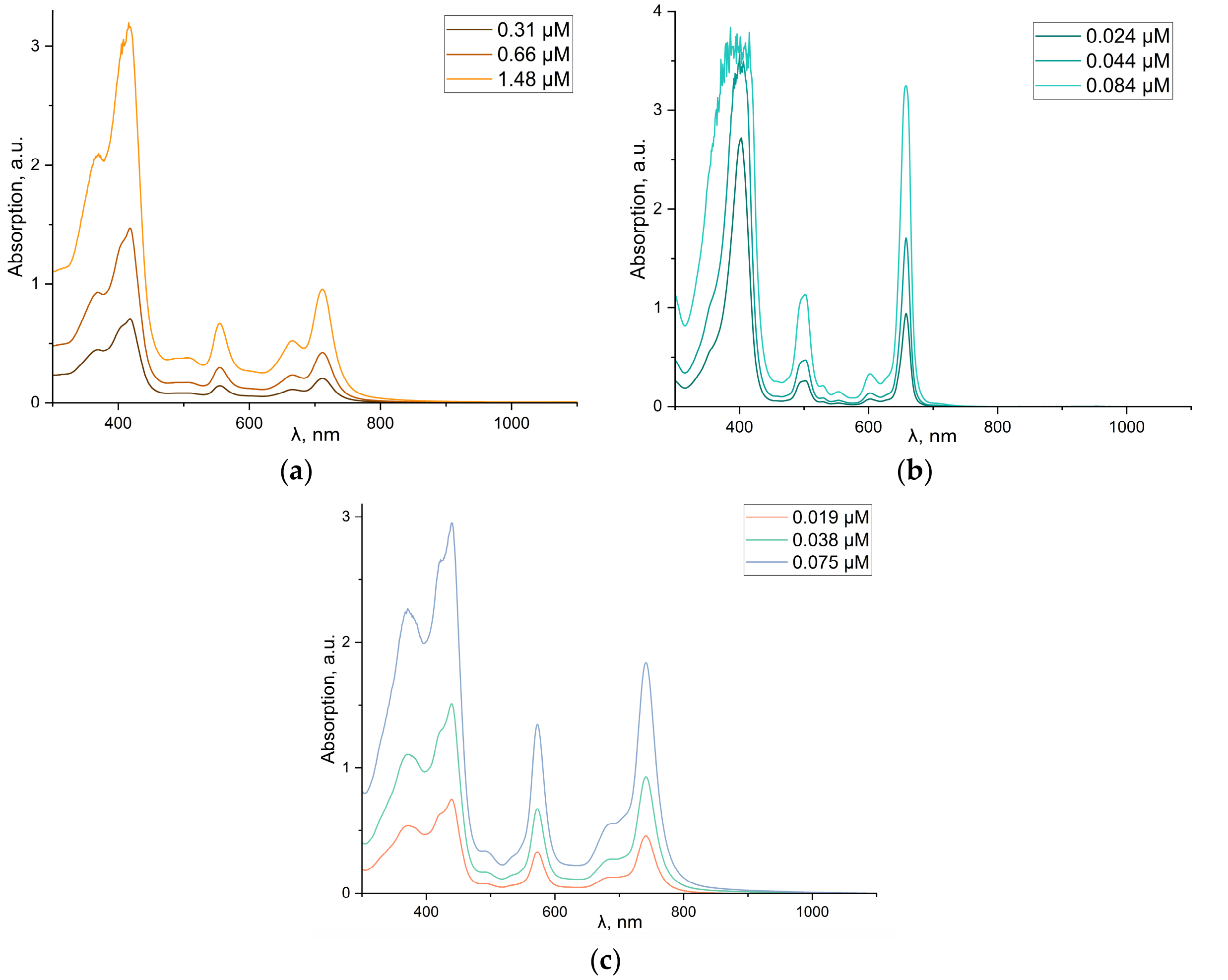

Figure S10: The electronic absorption spectra of compounds (**3**) (**a**), (**4**) (**b**) and (**5**) (**c**) at different concentrations in a water solution of 4% Kolliphor® ELP.

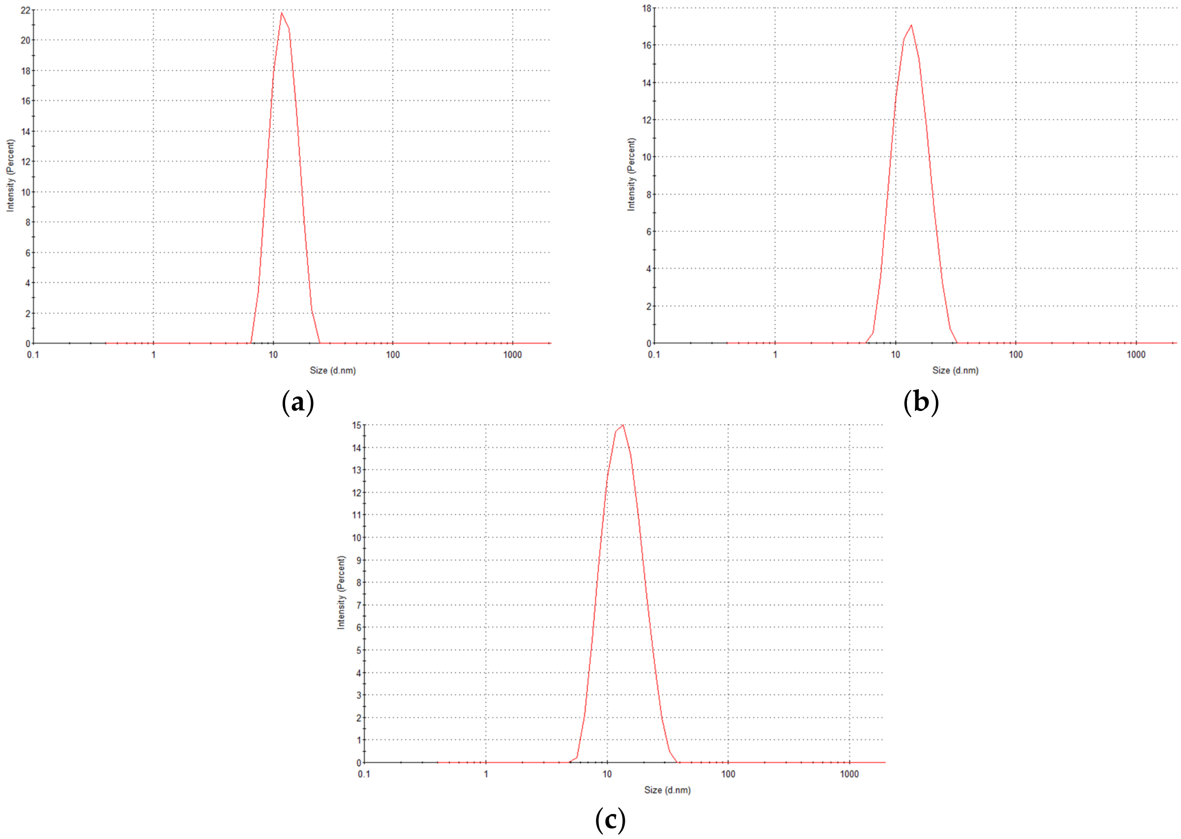

Figure S11: Particle size distribution by intensity using the DLS method for compounds (**3**) (**a**), (**4**) (**b**) and (**5**) (**c**) in a water solution of 4% Kolliphor® ELP.

Table S1: Shapes of molecular orbitals potentially involved in electronic transitions for compound (**4**).

Orbital	Energy, eV	View	
HOMO–5 149	-7.1491	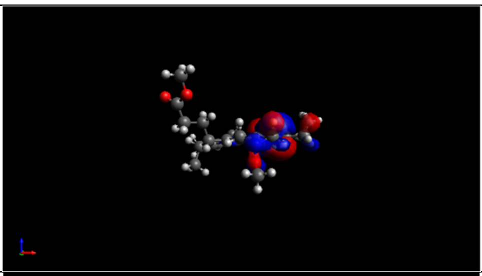	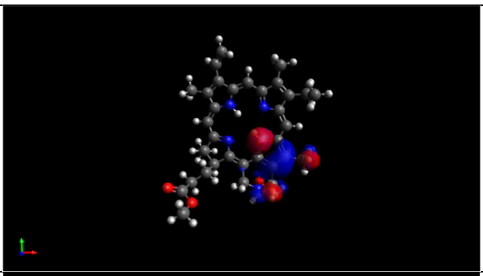
HOMO–4 150	-6.8976	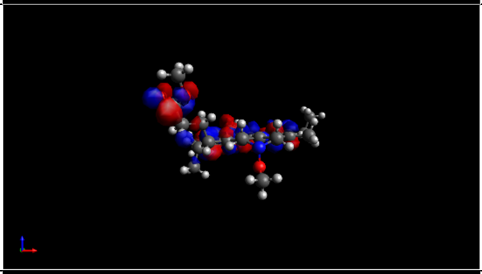	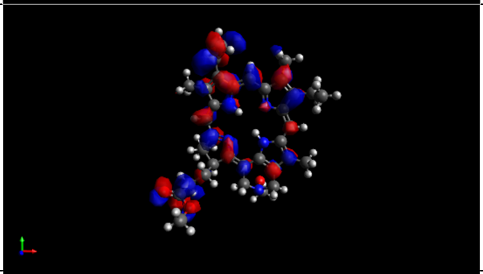
HOMO–3 151	-6.7717	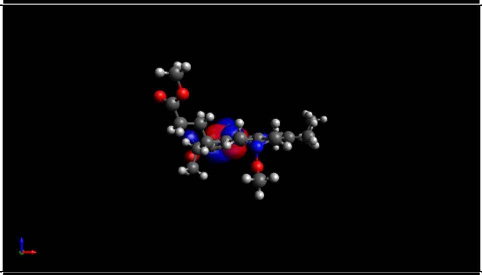	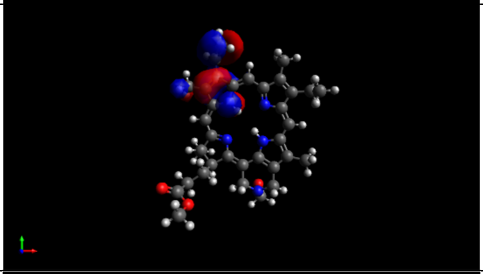
HOMO–2 152	-6.3170	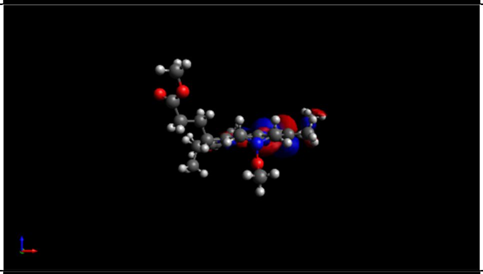	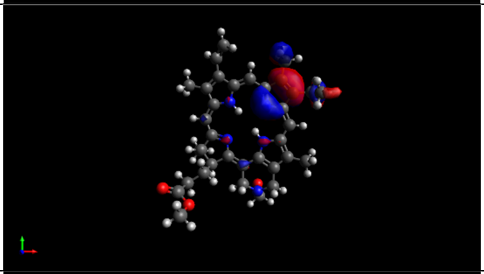
HOMO–1 153	-5.4314	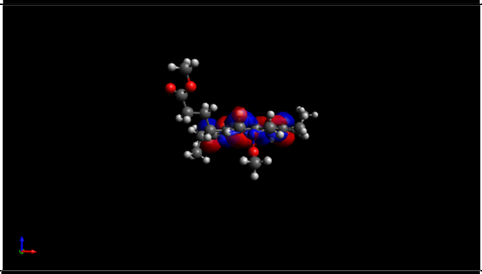	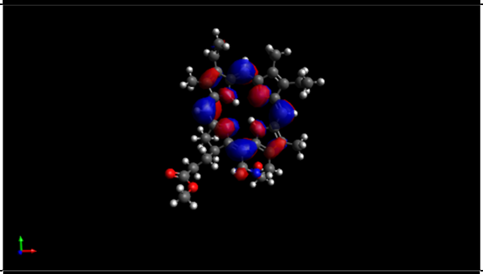
HOMO 154	-5.1080	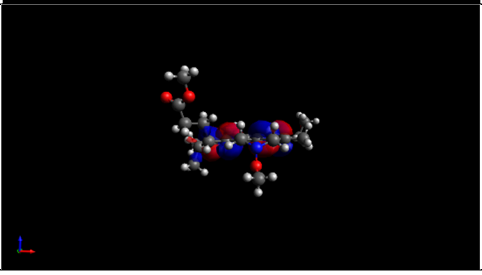	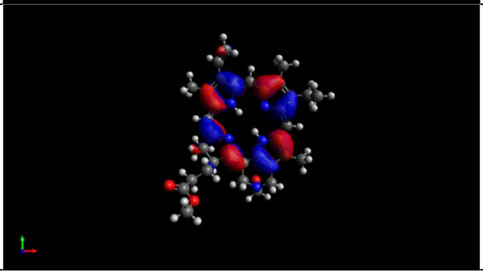

LUMO<br>155	-2.2234	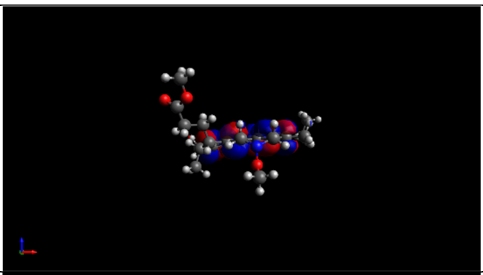	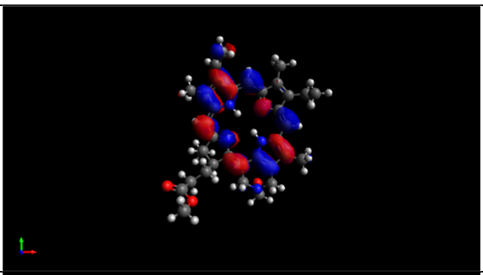
LUMO+1<br>156	-1.6723	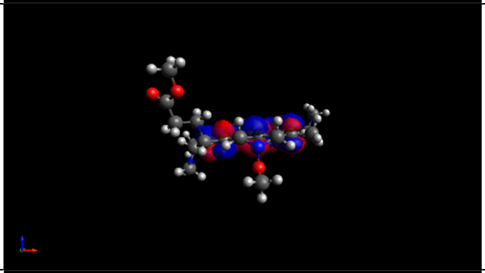	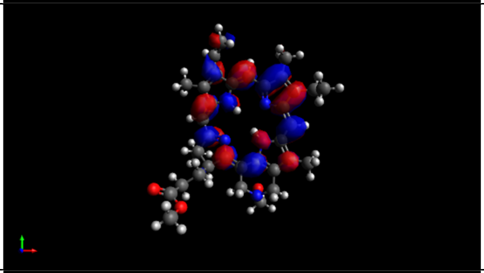
LUMO+2<br>157	-0.2014	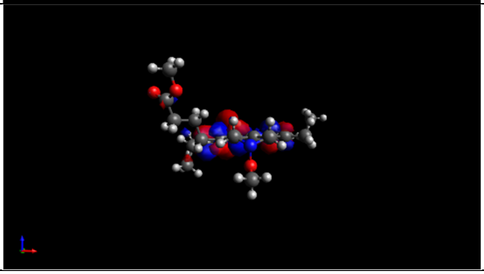	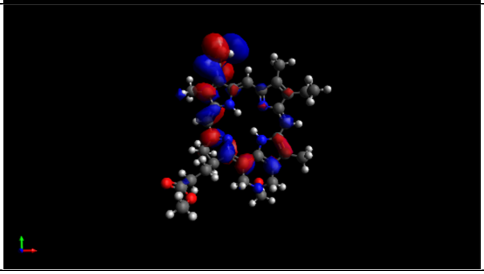
LUMO+3<br>158	0.2777	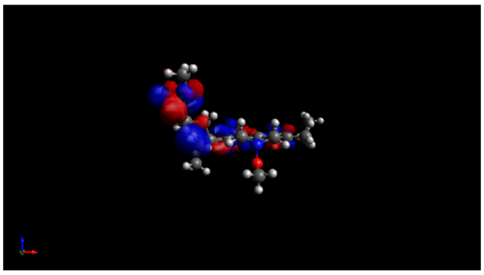	\ 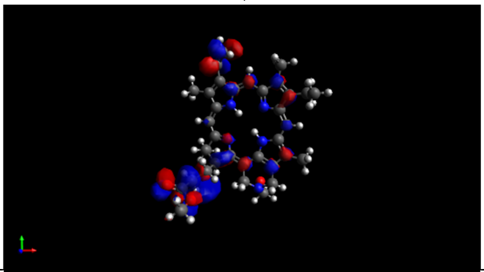
LUMO+4<br>159	0.4488	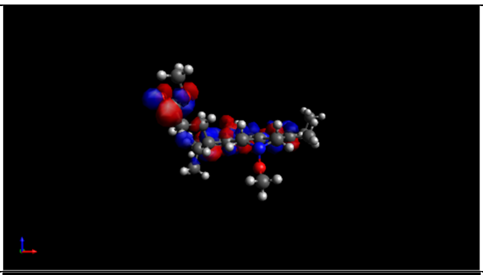	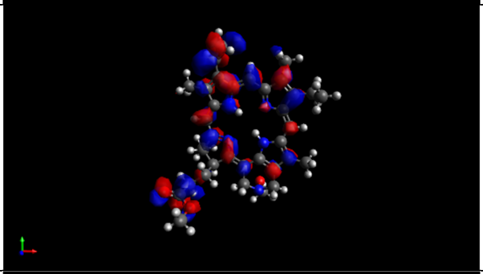
LUMO+5<br>160	0.7942	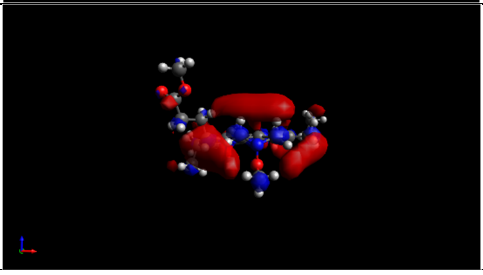	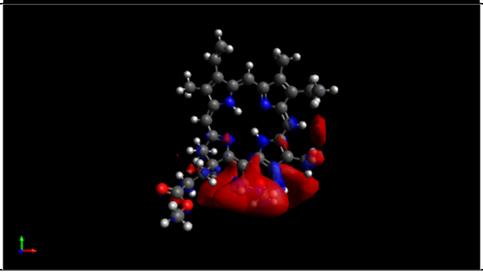

Table S2: Shapes of molecular orbitals potentially involved in electronic transitions for compound (**3**).

Orbital	Energy, eV	View	
HOMO–5 155	-7.5300	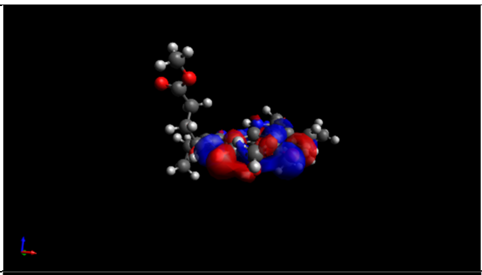	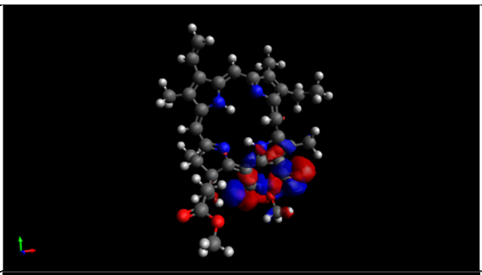
HOMO–4 156	-7.4270	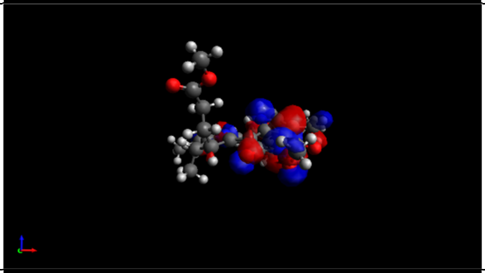	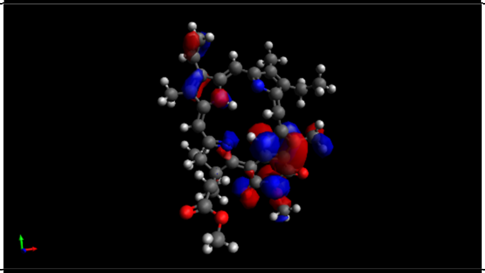
HOMO–3 157	-7.3881	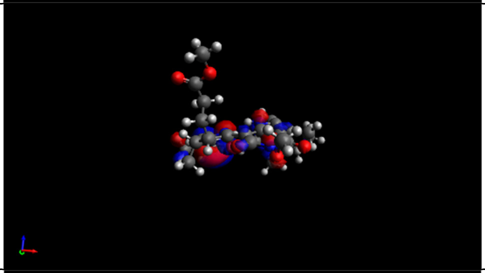	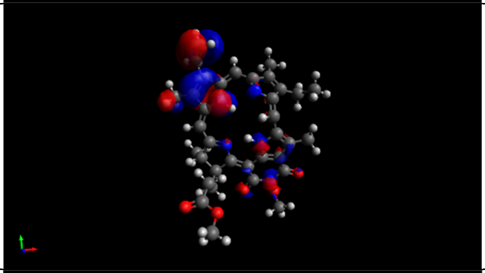
HOMO–2 158	-6.9787	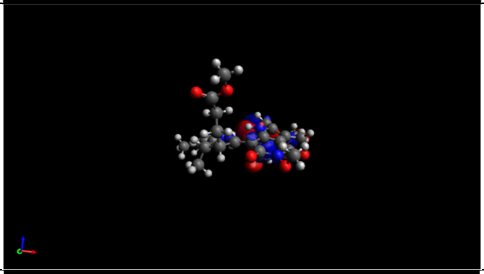	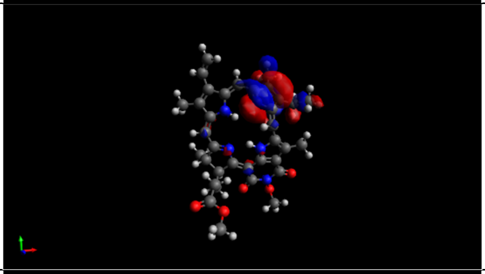
HOMO–1 159	-6.0041	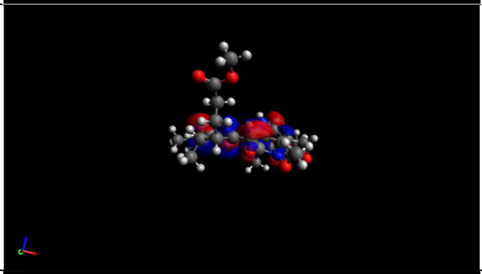	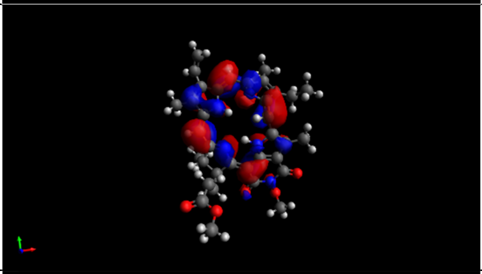
HOMO 160	-5.7678	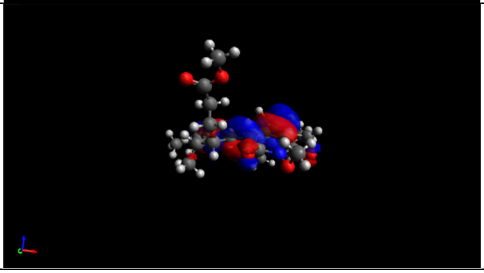	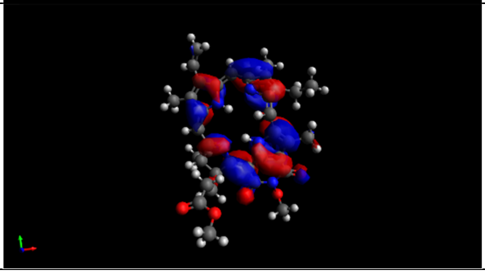

LUMO 161	-2.9932	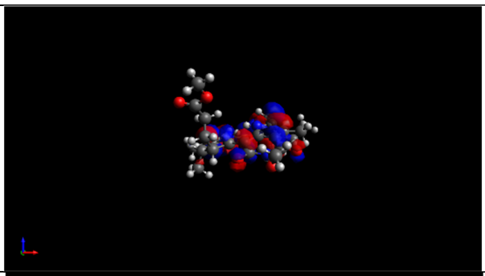	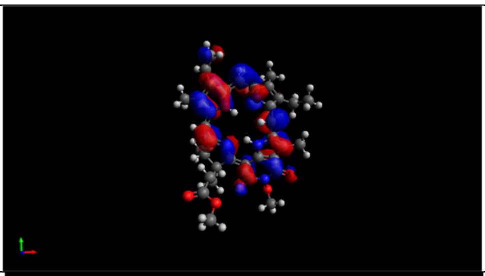
LUMO+1 162	-2.1886	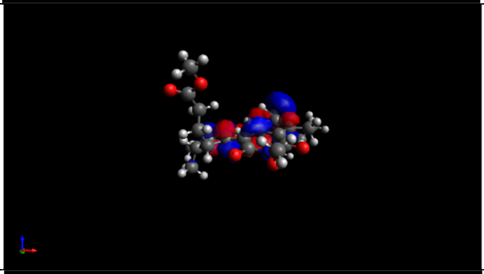	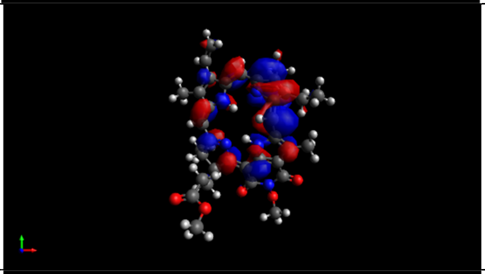
LUMO+2 163	-1.3790	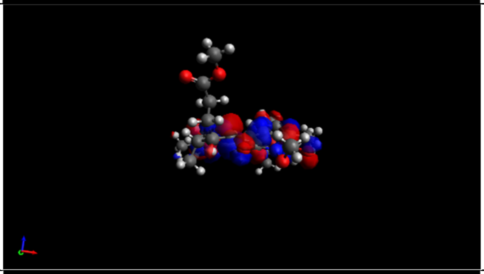	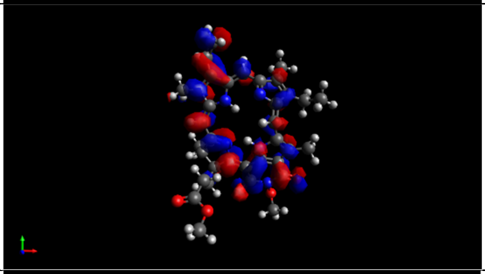
LUMO+3 164	-0.5218	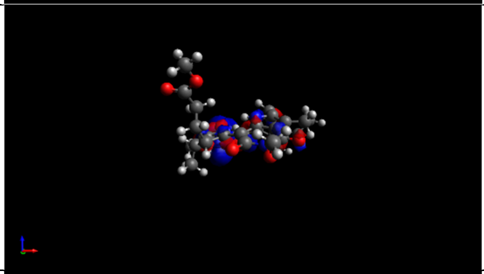	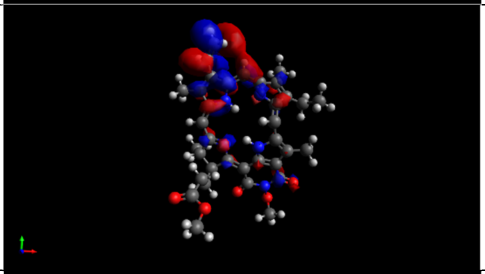
LUMO+4 165	0.0871	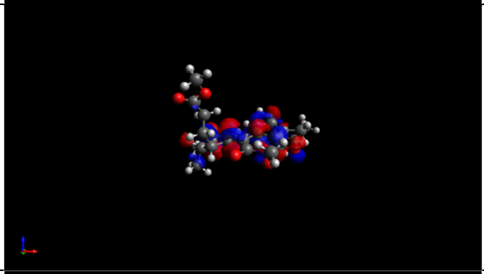	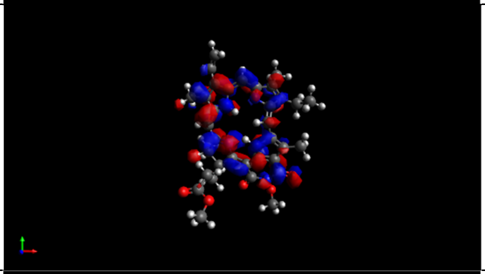
LUMO+5 166	0.4998	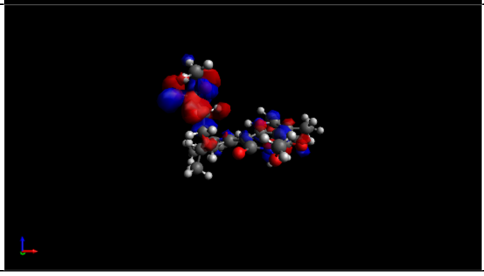	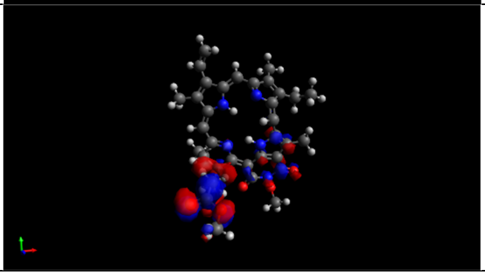

Table S3: Shapes of molecular orbitals potentially involved in electronic transitions for compound (**5**).

Orbital	Energy, eV	View	
HOMO–5 159	-7.2738	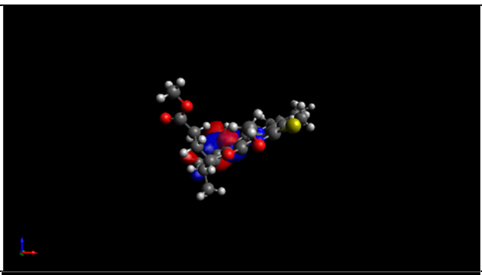	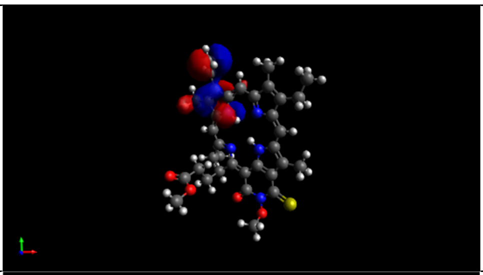
HOMO–4 160	-6.9234	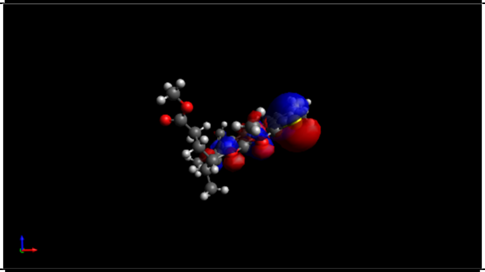	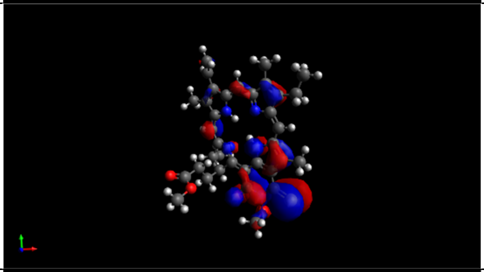
HOMO–3 161	-6.8230	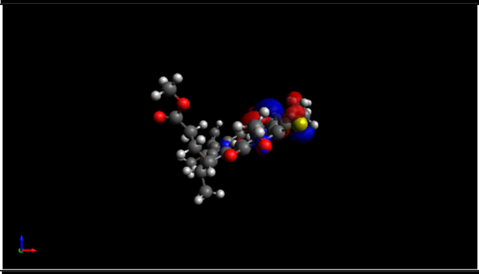	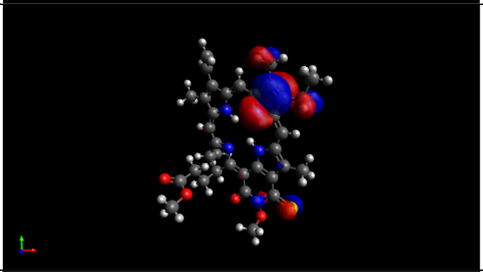
HOMO–2 162	-6.1386	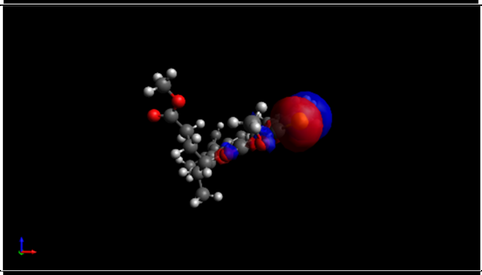	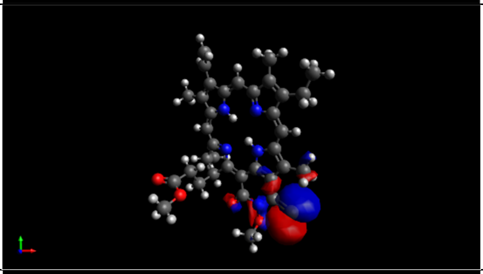
HOMO–1 163	-6.0767	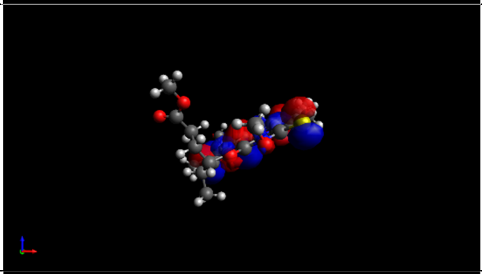	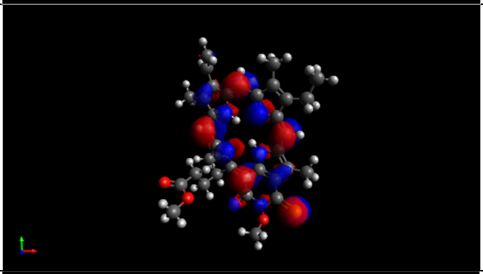
HOMO 164	-5.6570	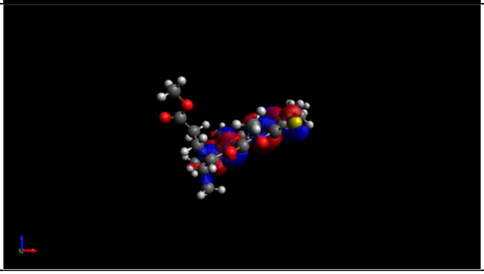	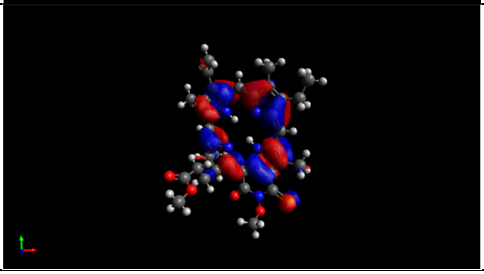

LUMO<br>165	-3.0895	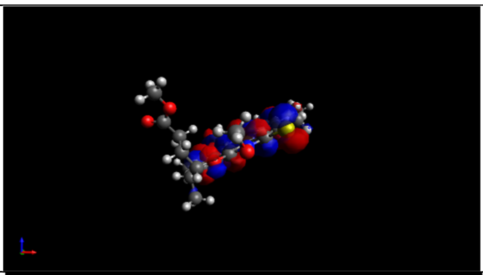	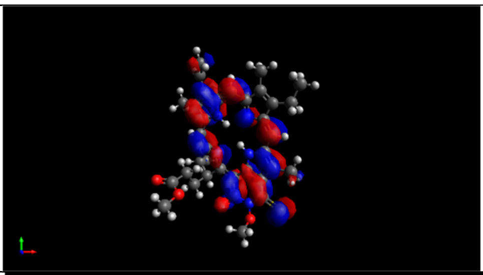
LUMO+1<br>166	-2.1533	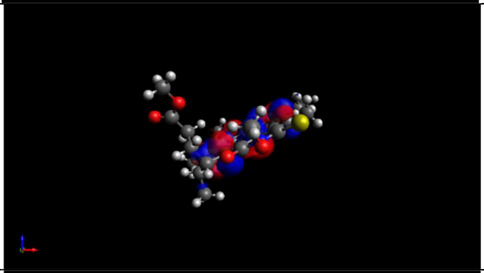	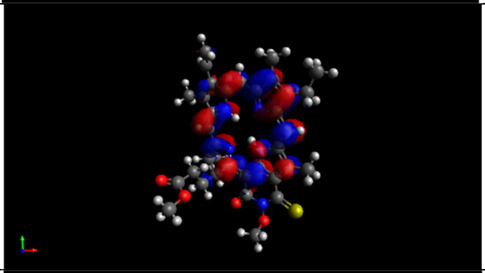
LUMO+2<br>167	-1.5780	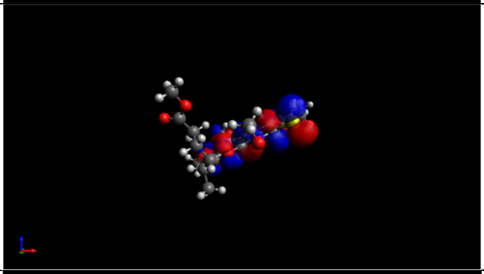	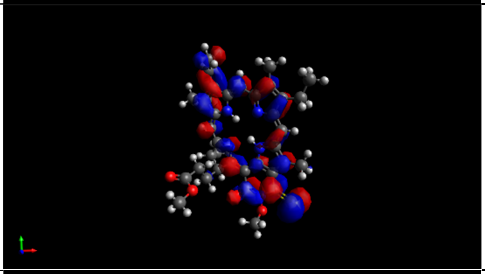
LUMO+3<br>168	-0.5159	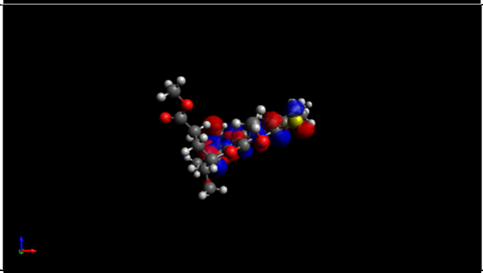	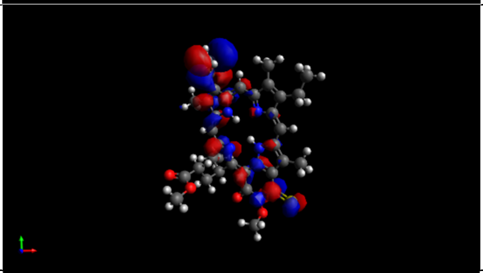
LUMO+4<br>169	-0.0184	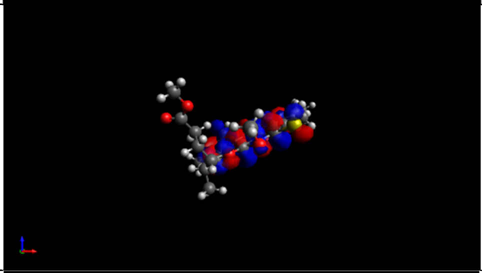	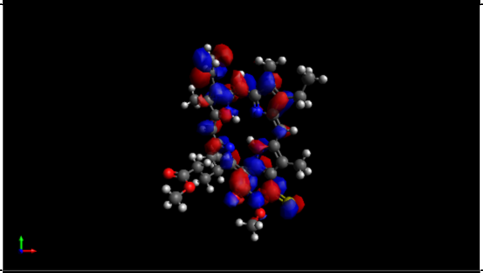
LUMO+5<br>170	0.4450	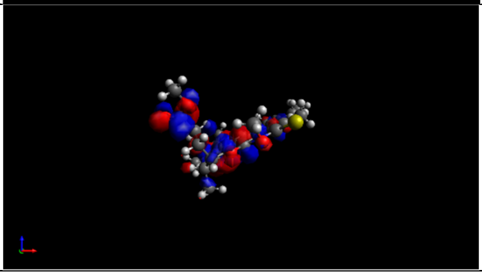	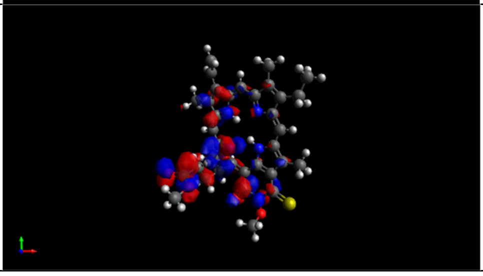

Table S4: Dependence of the percentage of inhibition of bacterial lines by the studied compounds (**3**)-(**5**) on the concentration with and without irradiation.

Staphylococcus aureus

	Concentration, μM	(**3**)	(**4**)	(**5**)
Irradiation	3.175	20.36 ± 2.07	83.83 ± 5.47	58.46 ± 1.32
	6.25	88.02 ± 0.96	91.74 ± 1.86	85.29 ± 3.14
	12.5	88.95 ± 0.64	93.08 ± 0.90	92.69 ± 2.24
	25	90.51 ± 1.06	96.21 ± 0.93	94.14 ± 0.94
	50	94.30 ± 1.30	97.17 ± 2.25	97.99 ± 0.47
No irradiation	3.175	16.02 ± 3.35	21.28 ± 4.30	25.62 ± 2.68
	6.25	22.95 ± 1.77	28.04 ± 2.50	31.19 ± 2.40
	12.5	29.59 ± 2.84	28.16 ± 1.01	32.84 ± 1.94
	25	34.43 ± 4.39	35.29 ± 2.96	34.29 ± 4.07
	50	36.18 ± 4.83	38.10 ± 3.04	36.17 ± 3.33

Pseudomonas aeruginosa

	Concentration, μM	(**3**)	(**4**)	(**5**)
Irradiation	3.175	31.73 ± 1.59	80.49 ± 4.59	54.32 ± 2.63
	6.25	85.28 ± 2.42	87.00 ± 1.95	71.29 ± 2.17
	12.5	90.01 ± 1.26	90.14 ± 0.91	89.51 ± 1.04
	25	92.16 ± 0.57	91.96 ± 1.38	92.32 ± 0.83
	50	93.55 ± 2.11	92.38 ± 1.87	97.41 ± 0.71
No irradiation	3.175	12.03 ± 2.00	21.49 ± 2.91	27.28 ± 1.40
	6.25	17.45 ± 1.05	28.27 ± 2.24	30.15 ± 1.21
	12.5	24.97 ± 1.47	28.25 ± 0.72	32.47 ± 1.63
	25	29.61 ± 1.16	35.52 ± 3.12	33.92 ± 4.24
	50	32.38 ± 1.38	38.46 ± 3.06	39.01 ± 3.46
